# Cell-Autonomous and Non-cell-autonomous Function of Hox Genes Specify Segmental Neuroblast Identity in the Gnathal Region of the Embryonic CNS in *Drosophila*

**DOI:** 10.1371/journal.pgen.1005961

**Published:** 2016-03-25

**Authors:** Henrike Becker, Simone Renner, Gerhard M. Technau, Christian Berger

**Affiliations:** Institute of Genetics, University of Mainz, Mainz, Germany; New York University, UNITED STATES

## Abstract

During central nervous system (CNS) development neural stem cells (Neuroblasts, NBs) have to acquire an identity appropriate to their location. In thoracic and abdominal segments of *Drosophila*, the expression pattern of Bithorax-Complex Hox genes is known to specify the segmental identity of NBs prior to their delamination from the neuroectoderm. Compared to the thoracic, ground state segmental units in the head region are derived to different degrees, and the precise mechanism of segmental specification of NBs in this region is still unclear. We identified and characterized a set of serially homologous NB-lineages in the gnathal segments and used one of them (NB6-4 lineage) as a model to investigate the mechanism conferring segment-specific identities to gnathal NBs. We show that NB6-4 is primarily determined by the cell-autonomous function of the Hox gene *Deformed* (*Dfd*). Interestingly, however, it also requires a non-cell-autonomous function of *labial* and *Antennapedia* that are expressed in adjacent anterior or posterior compartments. We identify the secreted molecule Amalgam (Ama) as a downstream target of the Antennapedia-Complex Hox genes *labial*, *Dfd*, *Sex combs reduced* and *Antennapedia*. In conjunction with its receptor Neurotactin (Nrt) and the effector kinase Abelson tyrosine kinase (Abl), Ama is necessary in parallel to the cell-autonomous *Dfd* pathway for the correct specification of the maxillary identity of NB6-4. Both pathways repress *CyclinE* (*CycE*) and loss of function of either of these pathways leads to a partial transformation (40%), whereas simultaneous mutation of both pathways leads to a complete transformation (100%) of NB6-4 segmental identity. Finally, we provide genetic evidences, that the Ama-Nrt-Abl-pathway regulates *CycE* expression by altering the function of the Hippo effector Yorkie in embryonic NBs. The disclosure of a non-cell-autonomous influence of Hox genes on neural stem cells provides new insight into the process of segmental patterning in the developing CNS.

## Author Summary

The central nervous system (CNS) needs to be subdivided into functionally specified regions. In the developing CNS of *Drosophila*, each neural stem cell, called neuroblasts (NB), acquires a unique identity according to its anterior-posterior and dorso-ventral position to generate a specific cell lineage. Along the anterior-posterior body axis, Hox genes of the Bithorax-Complex convey segmental identities to NBs in the trunk segments. In the derived gnathal and brain segments, the mechanisms specifying segmental NB identities are largely unknown. We investigated the role of Hox genes of the Antennapedia-Complex in the gnathal CNS. In addition to cell-autonomous Hox gene function, we unexpectedly uncovered a parallel non-cell-autonomous pathway in mediating segmental specification of embryonic NBs in gnathal segments. Both pathways restrict the expression of the cell cycle gene *CyclinE*, ensuring the proper specification of a glial cell lineage. Whereas the Hox gene *Deformed* mediates this cell-autonomously, *labial* and *Antennapedia* influence the identity via transcriptional regulation of the secreted molecule *Amalgam* (and its downstream pathway) in a non-cell-autonomous manner. These findings shed new light on the role of the highly conserved Hox genes during segmental patterning of neural stem cells in the CNS.

## Introduction

The *Drosophila* central nervous system (CNS) consists of 20 segmental units (neuromeres), the sizes and composition of which are specifically adapted to the functional requirements of the respective body parts in the head, thorax and abdomen. Thus, neural stem cells (called neuroblasts, NBs), although showing serial homologies among segments, generate distinct cell lineages in correspondence to their segmental assignment [1]. This segmental identity is conferred to NBs already in the embryonic neuroectoderm and persists during the generation of their larval and adult sublineages. Therefore, it is convenient to study mechanisms regulating the segmental specification of neural stem cells in the embryo.

Most studies on segmental specification of embryonic NBs in *Drosophila* were focused so far on thoracic (T1-T3) and abdominal (A1-A10) segments of the ventral nerve cord (VNC). Neuromeres T1-A7 are built by a stereotype pattern of approximately 30 NBs per hemisegment. Individual identities and serial homology of segmentally repeated NBs is reflected by position, marker gene expression [2, 3] and composition of their lineages [4–6]. However, some of the serially homologous NB-lineages exhibit specific differences between thoracic and abdominal segments, which are conveyed to NBs already in the neuroectoderm by Bithorax-Complex (Bx-C) Hox genes [7–9]. While thoracic identities seem to represent a ground state (T2, no input of Hox genes; [10]), identities of consecutive posterior segments are established by adding the function of Bx-C Hox genes *Ultrabithorax* (*Ubx*), *abdominal-A* (*abdA*) and *Abdominal-B* (*AbdB*), an evolutionary highly conserved phenomenon described as posterior dominance or prevalence of Hox genes [10–12]. The terminal abdominal neuromeres A8-A10 exhibit a progressively derived character regarding size and composition. In these segments, NB patterns and segmental identities are controlled by combined action of the Hox gene *AbdB* and the ParaHox gene *caudal* [13, 14].

The *Drosophila* head consists of seven segments (4 pregnathal and 3 gnathal) all of which contribute neuromeres to the CNS [15, 16]. The brain is formed by approximately 100 NBs per hemisphere, which have been individually identified and assigned to specific pregnathal segments [17, 18]. As judged from comparison of the combinatorial codes of marker gene expression only few brain NBs appear to be serially homologous to NBs in the thoracic/abdominal ventral nerve cord, reflecting the highly derived character of the brain neuromeres [19]. The connecting tissue between brain and the thoracic VNC consists of three neuromeres formed by the gnathal head segments named mandibular (mad), maxillary (max) and labial (lab) segment, but the number and identity of the neural stem cells and their lineage composition in these segments is still unknown. Compared to the thoracic ground state the segmental sets of gnathal NBs might be reduced to different degrees, but are thought to be less derived compared to the brain NBs. Therefore, to fully understand segmental specification during central nervous system development, it is important to identify the neuroblasts and their lineages in these interconnecting segments.

Assuming that most NBs in the gnathal segments still share similarities to thoracic and abdominal NBs, we searched in these segments for serially homologous NB-lineages, which are suitable for genetic analyses. Using the molecular marker *eagle* (*eg*), which specifically labels four NB-lineages in thoracic/abdominal hemisegments [20, 21] we identified three serial homologs (NB3-3, NB6-4 and NB7-3) in the gnathal region. To investigate the mechanisms conferring segmental identities, we focused on one of them, the NB6-4 lineage, which shows the most significant segment-specific modifications. Our analysis reveals a primary role of the Antennapedia-Complex (Antp-C) Hox gene *Deformed* (*Dfd*) in cell-autonomously specifying the maxillary fate of NB6-4 (NB6-4max). Surprisingly, we uncovered an additional, non-cell-autonomous function of the Antp-C Hox genes *labial* (*lab*, expressed anterior to *Dfd*) and *Antennapedia* (*Antp*, expressed posterior to *Dfd*) in specifying NB6-4max. In a mini-screen for downstream effectors we identify the secreted protein Amalgam (Ama) to be positively regulated by *lab*, *Dfd* and *Antp* and negatively regulated by the Antp-C Hox gene *Sex combs reduced* (*Scr*). Loss of function of *Ama* and its receptor *Neurotactin (Nrt*) [22–25] as well as the downstream effector kinase *Abelson tyrosine kinase* (*Abl*) [23] lead to a transformation of NB6-4max similar to *Dfd* single mutants. Thus, in parallel to the cell-autonomous role of *Dfd*, a non-cell-autonomous function of Hox genes *lab* and *Antp*, mediated via the Ama-Nrt-Abl pathway, is necessary to specify NB6-4max identity. Disruption of either of these pathways leads to a partial misspecification of NB6-4max (approx. 40%), whereas simultaneous disruption of both pathways leads to a complete transformation (approx. 100%) of NB6-4max to a labial/thoracic identity. We further show that both pathways regulate the expression of the cell cycle gene *CyclinE*, which is necessary and sufficient to generate labial/thoracic NB6-4 identity. Whereas Dfd seems to directly repress *CyclinE* transcription (similar to AbdA/AbdB in the trunk) [26], we provide indications that the Ama-Nrt-Abl pathway prevents *CyclinE* expression by altering the activity of the Hippo/Salvador/Warts pathway effector Yorkie (Yki).

## Results

### Identification of serially homologous Eagle-positive NB-lineages and variations of the Eagle-pattern in gnathal segments

**Fig 1:**
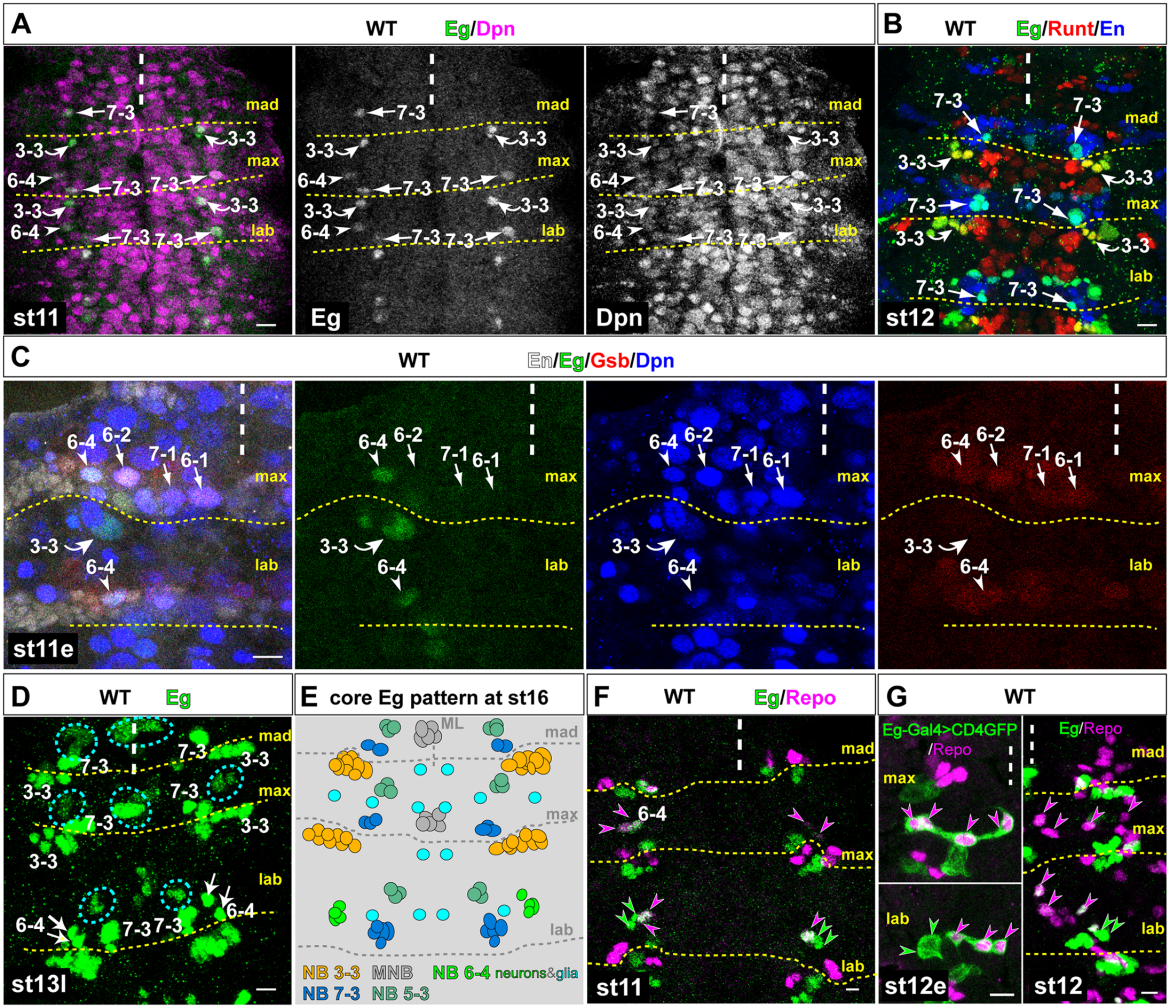
Description of the Eagle-positive Neuroblast-lineages in embryonic gnathal segments. (A) Identification of Eagle (Eg) expressing neuroblasts (NBs) in filet preparations of wild type gnathal CNS segments at embryonic stage 11. NBs were stained with Deadpan (Dpn) antibody (magenta); NBs 3–3, 6–4 and 7–3 were identified by co-staining with Eg antibody (green). Middle panel shows Eg channel in monochrome, right panel shows Dpn channel in monochrome. (B) NB3-3 can be identified by co-staining of Eg and Runt antibody (red), NB7-3 can be identified by co-staining of Eg and the segmental marker En (blue) and its typical dorso-medial position. (C) Co-staining of Eg (green), En (white), Dpn (blue) and Gooseberry (Gsb, red) antibody identifies NB6-4 and the presence of Gsb-positive/Eg-negative NB6-2, NB7-1 and NB6-1. (D) Additional cells (cyan circles) express Eg from stage 13 onwards. Progeny of MNB can be identified in the midline of mad and max segments and progeny of NB5-3 can be identified in all three segments. For identification of these cells see S1 Fig (E) Schematics of the core Eg expression pattern at stage 16. For simplicity extra Eg-positive cells are not highlighted. For complete pattern see S1 and S2 Figs. (F) Co-staining of Eg (green) with Repo (magenta) antibody reveals a pure glial NB6-4 lineage in maxillary segment and a mixed neuronal/glial lineage in the labial segment. (G) At stage 12 the maxillary NB6-4 lineage consists of four glial cells (magenta arrow heads), two medial and two lateral cell-body glial cells. The labial NB6-4 generates a dorso-lateral cluster of 4–5 neurons (green arrow heads) and three cell-body glial cells (magenta arrow heads) of which one is located ventro-laterally and two are in a ventro-medial position. The left panels shows *eg*Gal4 expressing UAS-*CD4*::*gfp* to show the clonal relation and to identify the four glial cells in the maxillary segment. In this and all following figures if not stated differently: anterior is up, dashed white line represents the midline, dashed yellow lines indicate segmental boundaries, mad, mandibular segment, max, maxillary segment and lab, labial segment, numbers indicate NBs or NB-lineages (e.g. 7–3 indicates NB7-3), magenta arrow heads indicate NB6-4 glioblast or its glial progeny cells, green arrow heads indicate NB6-4 neuronal daughter precursor or neuronal progeny cells, WT, wild type, st, stage, l, late, e, early, m, middle. Scale bar is 10 μm.

To identify serially homologous NBs in the gnathal segments, we used the transcription factor Eagle (Eg) as a marker. In the well-studied thoracic and abdominal segments Eg is expressed in four NBs and their lineages: NBs 2–4, 3–3, 6–4 and 7–3 [20, 21]. Staining WT embryos at stage11 (st11) with Eg and the NB-marker Deadpan (Dpn) (Fig 1A)[27] reveals a reduced number of Eg-positive NBs in gnathal segments. Using Eg and Dpn to identify NB2-4 [28] we observed, that NB2-4 is missing in all three gnathal segments (Fig 1). Co-staining of Eg and Runt at st12 identifies NB3-3 [29] in labial and maxillary segments, but not in the mandibular segment (Fig 1B). Eg and Engrailed (En) co-expression and its position in a typical dorso-posterior area of the En stripe indicates the presence of NB7-3 [20, 30] in all three gnathal segments (Fig 1A and 1B). NB6-4 is identifiable by combined expression of Eg, En and Gooseberry (Gsb)[31, 32] in labial and maxillary segments but is missing in the mandibular segment (Fig 1A and 1C).

Next we used Eg in combination with various other markers at st13 to st16 to describe the composition of the Eg-positive lineages (Figs 1D, 1E and S1). NB3-3 generates a lineage of approximately 9 neurons, which is less than described for thoracic/abdominal lineages with 10–13 cells [6]. Among these we observed 7 neurons expressing Even-skipped (Eve) in maxillary and labial segments (S1A Fig), compared to 5 (on average) in the thoracic and 9 (on average) in the abdominal lineages [6, 14, 33].

The early NB7-3 lineage consist of six to seven cells, expressing the marker Eyeless (Ey; Figs 1E and S1B) [13, 34]), but due to segment-specific cell death at later stages [35, 36] the cell numbers in each segment are distinct. At st16 we find the mandibular NB7-3mad lineage to consist of two cells, the maxillary NB7-3max of three cells and the labial NB7-3lab of three to five cells (Fig 1E). In comparison, in T1 and T2 the late embryonic NB7-3 lineage consists of four, in T3-A7 of three, in A8 of two or three, and in A9 and A10 NB7-3 is not formed [14, 36].

NB6-4 generates a mixed neuronal/glial lineage (4–5 neurons, 3 glia) in labial segments as revealed by staining against Eg and the glial marker Repo (Fig 1E–1G)[37]. Its division pattern is identical to thoracic NB6-4, in which the first division separates a glial from a neuronal daughter precursor and factors like Prospero or *glia cells missing* (*gcm)* (S1C Fig) are asymmetrically localized to the glial progenitor [7, 38–40]. In contrast, similar to NB6-4 in abdominal segments [6], NB6-4max is a pure glioblast, generating four glial progeny cells (Fig 1E–1G).

To our surprise, we observed more Eg-positive cell clusters in gnathal segments from st12 onwards (Figs 1D, 1E, S1 and S2), that we could identify as progeny of the Midline Neuroblast (MNB, in mad and max segments), progeny of the NB5-3 (in all three segments) [41–45] and from stage 14 onwards we could identify Eg-positive late progeny of NBs 6–2, 4–3, 4–4 and 5–6 (in mad and max segments). Further details can be found in Figs 1D, 1E, S1 and S2.

Taken together, we show that (1) despite of its complex expression pattern in the late embryo, Eg is a reliable marker to identify serially homologous NBs and their progeny in gnathal segments, (2) whereas NB2-4 is missing in all gnathal segments, NB3-3 and NB6-4 are present in labial and maxillary segments, and NB7-3 is present in all three gnathal segments, (3) segment-specific differences occur in the NB7-3 lineage with regard to neuronal cell numbers, and in the NB6-4 lineage with regard to cell types: While NB6-4lab gives rise to a mixed neuronal/glial lineage corresponding to the thoracic homologs, NB6-4max is a pure glioblast corresponding to the abdominal homologs.

In the following we will focus on the NB6-4 lineage to elucidate the mechanism conferring segmental specificities in the gnathal CNS.

### *Deformed* and *Sex combs reduced* are co-expressed in NB6-4max, and NB6-4lab expresses *Antennapedia*

**Fig 2:**
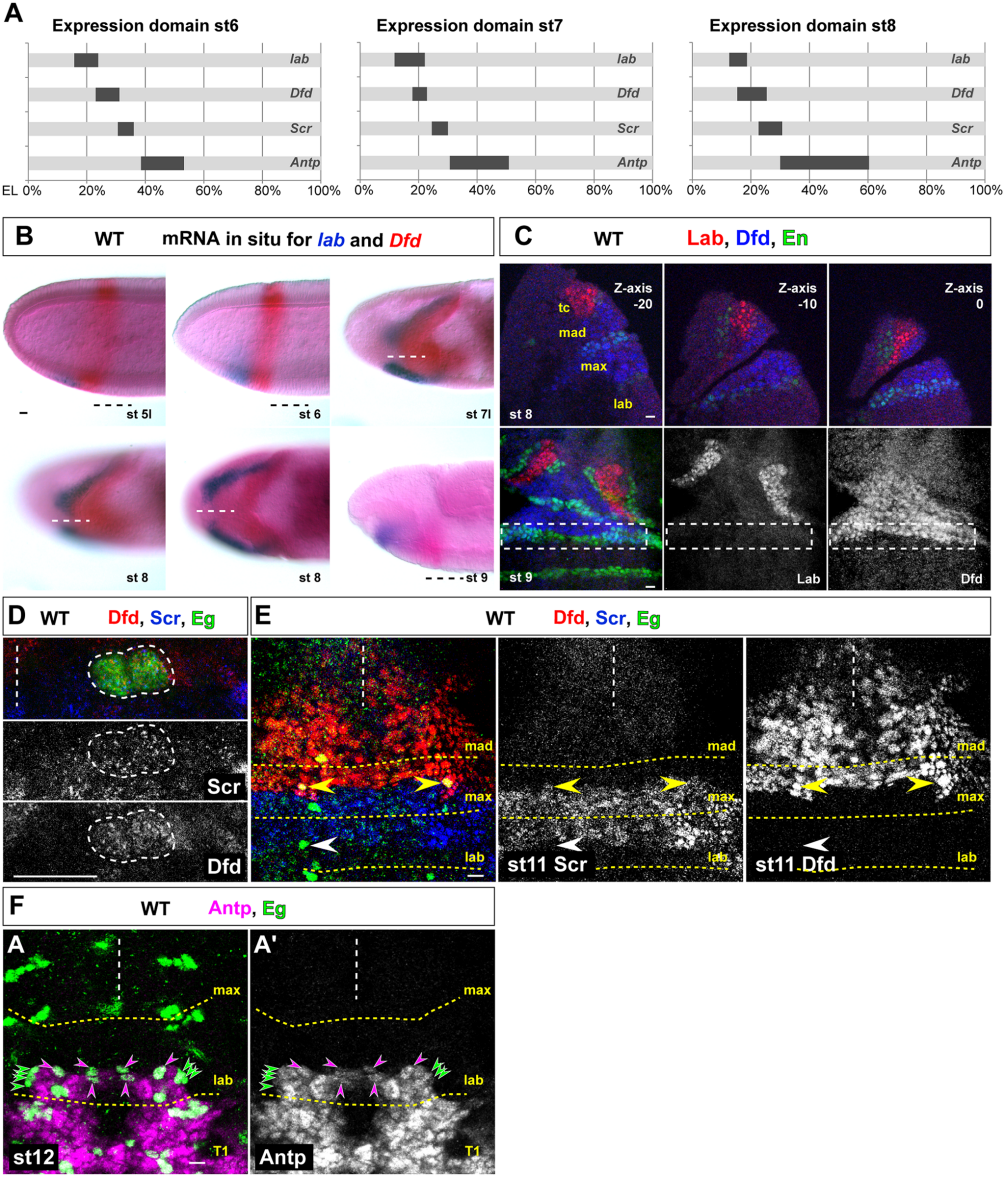
Expression pattern of Hox genes in embryonic gnathal segments. (A) Schematic representation of the average mRNA expression domains (in % of whole egg length, EL) of the Hox genes *labial* (*lab*), *Deformed* (*Dfd*), *Sex combs reduced* (*Scr*) and *Antennapedia* (*Antp*) in stage 6, 7 and 8. For original staining see S3 Fig. (B) Double *in-situ* hybridization to visualize expression pattern of *lab* (blue) and *Dfd*-mRNAs (red) at stage 5l to 9. No co-expression can be observed. Anterior to the left. Dashed line marks the ventral midline. (C) Antibody staining in the maxillary segment to visualize the protein expression pattern of Lab (red) and Dfd (blue) in relation to the Engrailed (En, green) domain from which NB6-4 segregates. No co-expression can be observed in stage 8 or 9. Upper panel shows three successive focal planes of a lateral view (anterior up, ventral to the right) of a whole mount embryo (Z-axis from -20, -10 to 0 μm). Tc, tritocerebrum. Lower panel shows filet preparation (anterior up, ventral view). The dash-lined box indicate the En-positive domain, from which NB6-4 delaminates; no expression of Lab can be observed. (D) Dfd (red) and Scr (blue) are co-expressed in the maxillary NB6-4 (Eg, green); two-cell stage shown at late stage 11. (E) NB6-4 expresses Dfd (red) and Scr (blue) in the maxillary segment (yellow arrow head), whereas NB6-4 in the labial segment lacks both of them (white arrow head). (F) All cells of the NB6-4 lineage in the labial segment express Antp (magenta). T1, first thoracic segment. Scale bar is 10 μm.

Since it was shown for thoracic/abdominal segments that Bx-C Hox genes convey the regional specification on the progenitor level [9, 13, 46], we next analyzed the mRNA expression pattern of Antp-C genes *labial* (*lab*), *Deformed* (*Dfd*), *Sex combs reduced (Scr*) and *Antennapedia* (*Antp*) in the presumptive neuroectoderm of maxillary and labial segments from st4 onwards. The widths of the mRNA stripes were measured in whole mount embryos using the distance from the anterior pole in relation to the length of the whole egg (EL; see material and methods; S3 Fig). Fig 2A shows the average expression domains in st6, st7 and st8 schematically. In contrast to earlier reports [47, 48], we could detect an overlap of *Dfd* and *Scr*-mRNA expression from st6 onwards (due to tissue invagination in the cephalic furrow at st7 this overlap is hardly visible, compare to Fig 2B) in the presumptive neuroectoderm of NB6-4max. The presumptive neuroectoderm of NB6-4lab shows *Antp*-mRNA expression only. In st7/8 it seemed that *lab*-mRNA expression was overlapping with *Dfd*-mRNA (Fig 2A), but close investigation showed, that due to the morphogenetic movements during cephalic furrow formation two epithelial sheets are overlapping but no co-staining can be found in cells of the neuroectoderm from which NB6-4max will delaminate (Fig 2B). We confirmed our observations using antibody staining against Lab, Dfd and the segmental marker En and could not observe a co-staining of Lab and Dfd in the maxillary stripe at st8/9, which is prior to NB6-4 delamination from the neuroectoderm (Fig 2C, see also [49]). Next we performed antibody staining for Dfd, Scr and Antp together with Eg and could indeed verify that NB6-4max co-expresses Dfd and Scr (Fig 2D and 2E), whereas NB6-4lab expresses Antp (Fig 2F) and no Scr. We also tested for Proboscipedia (Pb) expression and could observe single cell clusters distributed over the whole CNS [47] but we could not detect Pb expression in NB6-4 in gnathal segments (S4O Fig).

Therefore, of all Antp-C genes tested on mRNA and protein level, Dfd and Scr are expressed in NB6-4max, whereas NB6-4lab expresses Antp. *labial* and *Antp* are neither expressed in the maxillary neuroectoderm nor in the neuroblast NB6-4max itself.

### Segment specificity of NB6-4max is primarily determined by *Deformed*, but influenced in a parallel mechanism by *labial* and *Antennapedia*

**Fig 3:**
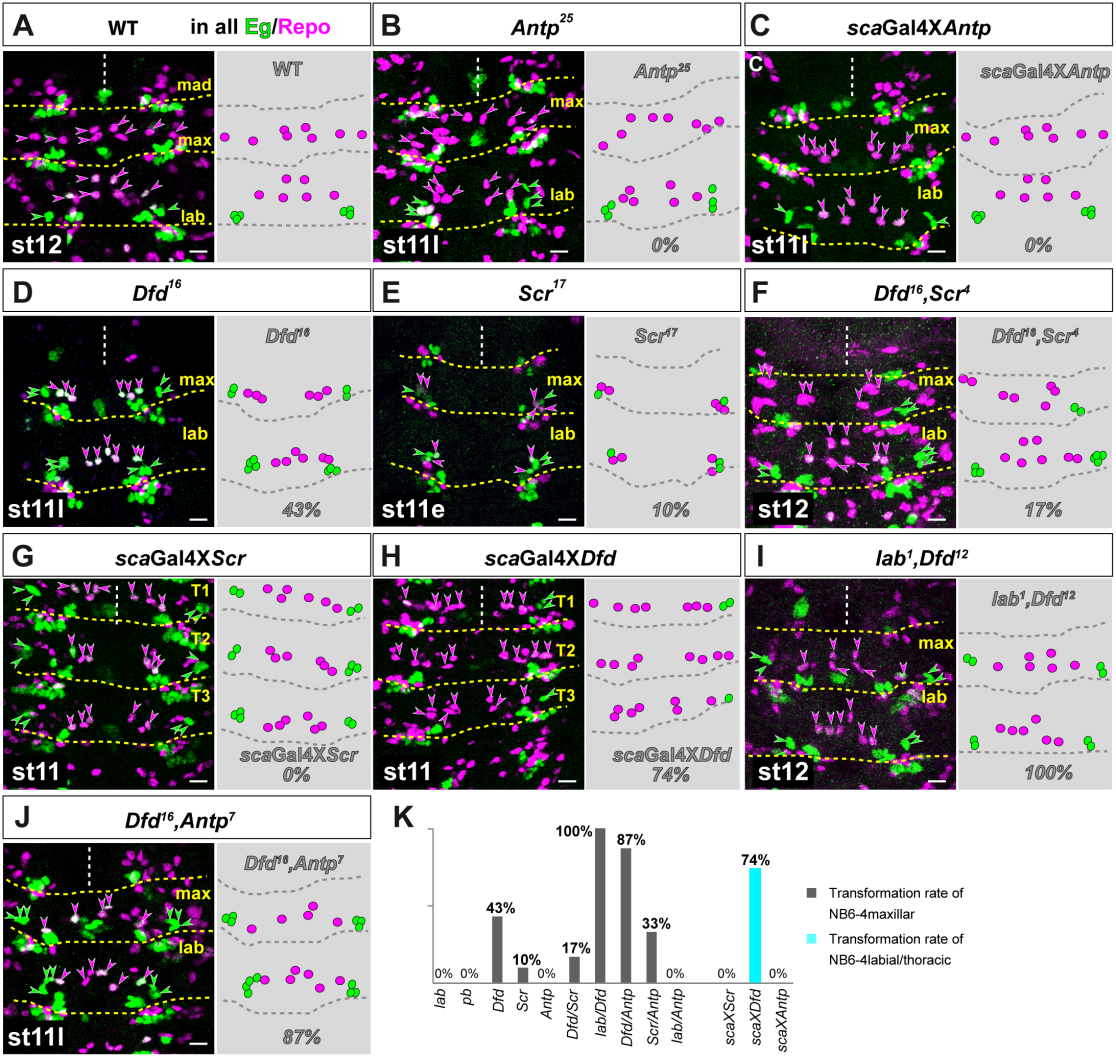
Segment specificity of NB6-4max is primarily determined by *Deformed*, but influenced in a parallel mechanism by *labial* and *Antennapedia*. For all images (A-J): Magenta arrow heads or spheres depict NB6-4-derived glial cells, green arrow heads or spheres depict NB6-4-derived neuronal cells. Segments are indicated. Right side shows an illustration of the composition of the NB6-4 lineage in the respective segments. Percentage gives transformation rate of NB6-4. (A) Wild type lineages of NB6-4 in maxillary and labial segments. NB6-4 generates 4 glial cells per maxillary hemisegment and 3 glial cells and a neuronal cluster per labial hemisegment. (B) In *Antennapedia* (*Antp*^*25*^) mutants NB6-4 cell fate is not altered in maxillary or labial segments. (C) Ectopic expression of *Antp* using the *scabrous*-Gal4 (*sca*Gal4) line does not alter NB6-4 cell fates in maxillary or labial segments. (D) Mutation in *Deformed* (*Dfd*^*16*^) leads to a transformation of NB6-4max glioblast into a neuroglioblast in 43% of all hemisegments. (E) Mutation in *Sex combs reduced* (*Scr*^*17*^) leads to a transformation of NB6-4max glioblast into a neuroglioblast in 10% of all hemisegments. (F) Double mutation of *Dfd*^*16*^ and *Scr*^*4*^ leads to a reduction of the transformation rate (17%) compared to *Dfd* single mutants (Fig 3D, 43%). Dfd and Scr do not act synergistically. (G) Ectopic expression of *Scr* using the *scabrous*-Gal4 (*sca*Gal4) line does not alter NB6-4 cell fates in thoracic or labial (see S4A Fig) segments. (H) Ectopic expression of *Dfd* using the *scabrous*-Gal4 (*sca*Gal4) line transforms thoracic and labial (see S4B Fig) NB6-4 into a maxillary fate producing only glial cells in 74% of all thoracic hemisegments. (I) Double mutation of *lab*^*1*^ and *Dfd*^*12*^ leads to a complete transformation of NB6-4max glioblast fate into a labial/thoracic neuroglioblast fate in 100% of all hemisegments. (J) Double mutation of *Dfd*^*16*^ and *Antp*^*7*^ leads to a nearly complete transformation of NB6-4max glioblast fate into a labial/thoracic neuroglioblast fate in 87% of all hemisegments. (K) Summary diagram of transformation rates in NB6-4maxillar (grey bars) or NB6-4labial/thoracic (cyan bars) in the respective mutant or ectopic expression situation. Transformation rates are given in percentage of analyzed hemisegments (number of hemisegments see text). Scale bar is 10 μm.

To analyze the influence of Hox genes on the segmental identity of NB6-4 in gnathal segments, we next tested different single or double mutants and overexpression of Antp-C genes (Fig 3A shows wild type). We first analyzed *Antp* loss-of-function (LoF) alleles *Antp*^*25*^ and *Antp*^*11*^ and could not observe a change in the segmental identity of both NB6-4max and NB6-4lab (*Antp*^*25*^, n = 50 maxillary hemisegments (mHs); *Antp*^*11*^, n = 50 mHs; Fig 3B shows *Antp*^*25*^; since different mutant Hox gene alleles revealed similar phenotypes we show in this and the following experiments the results for only one allele). Furthermore, overexpression of *Antp* has no effect on the NB6-4 lineage identity in any segment (n = 50 mHs; Fig 3C). Therefore, similar to the thoracic NB6-4 lineages [46] the labial NB6-4 identity represents a ground state, which does not require Hox gene function.

Next, we tested the LoF alleles *Dfd*^*16*^, *Dfd*^*12*^ and *Dfd*^*11*^ for their impact on NB6-4max. Double staining against Eg and the glial cell marker Repo revealed that loss of *Dfd* leads to a homeotic transformation of NB6-4max into a mixed lineage comprising neurons and glial cells (corresponding to NB6-4lab lineage) in approximately 43% of all hemisegments (*Dfd*^*16*^ n = 650 mHs, *Dfd*^*12*^ n = 650 mHs; *Dfd*^*11*^ n = 650 mHs; Fig 3D shows *Dfd*^*16*^). Since NB6-4max expresses Dfd and Scr, we analyzed two LoF alleles of *Scr* (*Scr*^*17*^ (n = 500 mHs) and *Scr*^*11*^ (n = 500 mHs)). In 10% of the mutant hemisegments NB6-4max produces neurons and glial cells (Fig 3E shows *Scr*^*17*^). As this suggests that *Dfd* and *Scr* might act synergistically, we analyzed the effect of double LoF of *Dfd*^*16*^ and *Scr*^*4*^ (n = 500 mHs). Surprisingly, instead of an increase in the transformation rate we could observe a reduced transformation rate in NB6-4max (17%; Fig 3F) compared to single *Dfd* LoF situation (Fig 3D, 43%). In addition, whereas ectopic expression of *Scr* (n = 50 mHs) has no effect on labial (S4A Fig) and thoracic NB6-4 (Fig 3G), the ectopic expression of *Dfd* leads to a transformation of labial (S4B Fig) and thoracic NB6-4 (74% of all hemisegments (n = 160 mHs), Fig 3H) towards a maxillary identity with four glial cells and no neurons. Thus, *Dfd* seems to be the major Hox gene influencing the NB6-4max identity cell-autonomously. Since loss of function only leads to a partial transformation rate (43%), we wondered if any of the other Antp-C genes, although not expressed in NB6-4, might be involved in specifying NB6-4max. Surprisingly, we observed an increase of the transformation rate in double LoF of *lab*^*1*^*/Dfd*^*12*^ (100%, n = 650 mHs, Fig 3I) and *Dfd*^*16*^/*Antp*^*7*^ (87%, n = 650 mHs, Fig 3J) compared to 43% in single *Dfd* mutants (Fig 3D). None of the single mutant alleles of *lab*^*4*^ (n = 50 mHs, S4C Fig) or *Antp*^*25*^ (see above) or the double LoF of *lab*^*1*^ and *Antp*^*25*^ (n = 100 mHs, S4D Fig) showed any change of the identity of NB6-4max. To show that loss of Hox gene function leads to a transformation of the maxillary NB6-4 on the progenitor level, we stained *lab*^*1*^*/Dfd*^*12*^ mutants for Eg and *gcm*-mRNA. Whereas the wild type NB6-4max distributes *gcm*-mRNA equally to both daughter cells (see S1C Fig), the transformed NB6-4 in maxillary segments of *lab*^*1*^*/Dfd*^*12*^ mutants shows an asymmetrically distribution of *gcm*-mRNA to the glial sublineage only (S4E Fig), like wild type labial (see S1C Fig) or thoracic [7, 38–40] progenitors do. Furthermore, the loss of *Scr* together with *Antp* increased the transformation rate to 33% (n = 100 mHs; S4F Fig) compared to 10% for *Scr* LoF alone.

Since neither *Antp* nor *lab* is expressed in wild type NB6-4max, we next investigated if the expression pattern of Hox genes is altered in mutant background. In *Dfd* mutants we could not detect any changes in Hox gene expression (single or double mutants, S4G–S4N Fig) with the exception of a slight extension of the *lab* expression domain towards the anterior mandibular segment (see also [50]) and a loss of Scr protein in NB6-4max. This protein loss seems to be due to a translational repression of Scr, since we detected normal expression of *Scr*-*m*RNA in *Dfd* mutants (S4H Fig).

Taken together, our expression and mutant analyses reveals a cell-autonomous function of Dfd and Scr in specifying NB6-4max (Fig 3K). Dfd seems to be the major cell-autonomously acting Hox gene in NB6-4max, since only ectopic expression of *Dfd*, but not *Scr*, can transform NB6-4lab/thoracic into a maxillary identity. Surprisingly, we detected a non-cell-autonomous effect of the more anterior expressed *lab* and the more posterior expressed *Antp* gene. Since these two genes are normally not expressed in NB6-4max nor in the neuroectodermal primordium and we did not observe mis-regulation of other Hox genes in NB6-4max in any Antp-C-mutant situation, we conclude that Lab and Antp act in a non-cell-autonomous manner to contribute to the determination of the maxillary segmental specificity of NB6-4.

To identify potential effectors downstream of the non-cell-autonomously acting Hox genes *lab* and *Antp* in NB6-4max specification, we next conducted a mini-screen.

### A mini-screen for novel regulators of NB6-4max specification identifies the secreted protein Amalgam as potential Hox gene target

**Fig 4:**
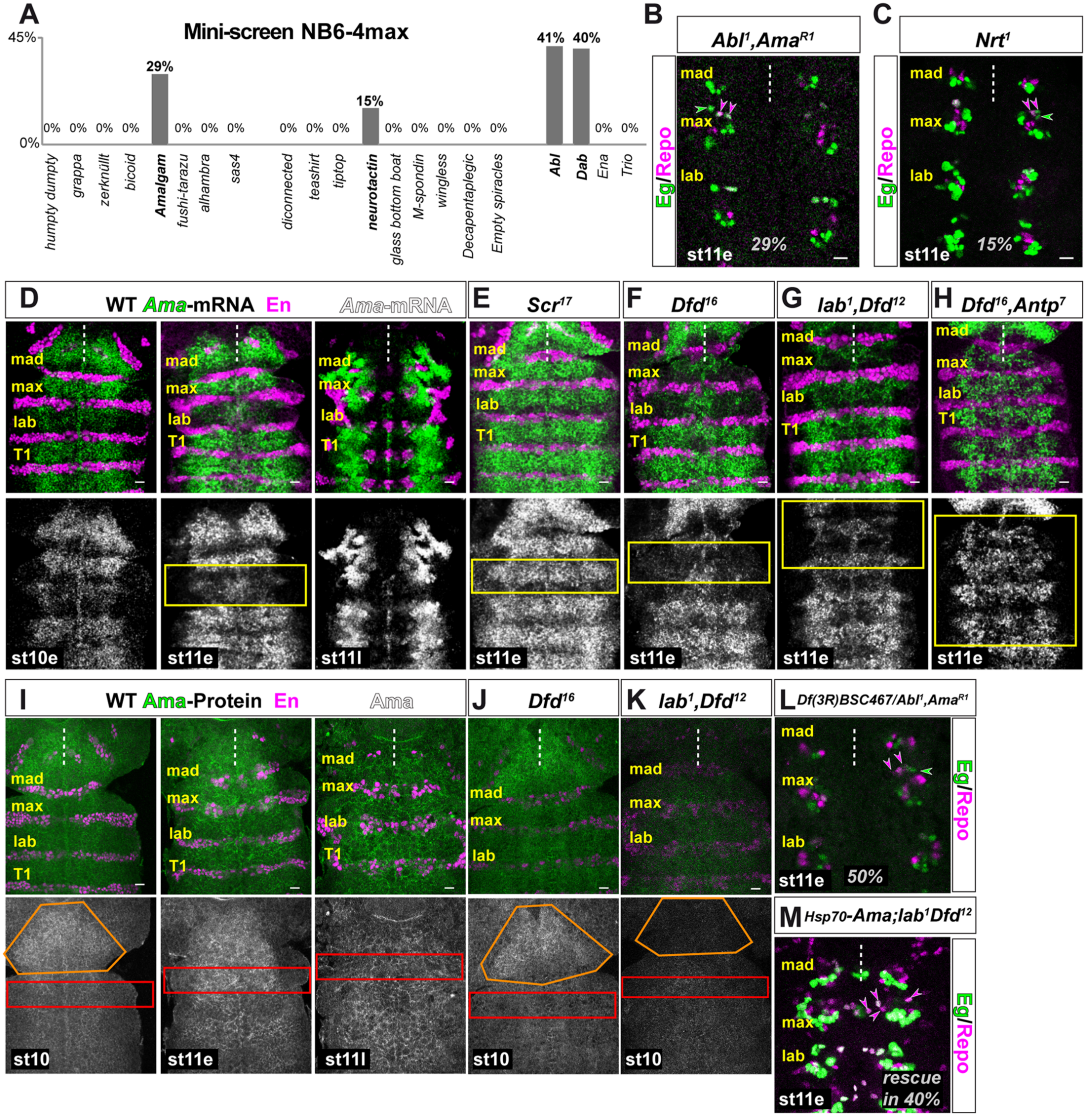
A mini-screen for novel regulators of NB6-4max specification identifies the secreted protein Amalgam as potential Hox target. (A) Mutant mini-screen for potential Hox target genes influencing NB6-4max segmental specification. Transformation rates of NB6-4max are given in percentage of analyzed maxillary hemisegments (number of hemisegments see text). (B) Double mutation of *Abl*^*1*^ and *Ama*^*R1*^ leads to a transformation of NB6-4max into a neuroglioblast in 29% of all hemisegments. (C) Mutation of *Nrt*^*1*^ leads to a transformation of NB6-4max into a neuroglioblast in 15% of all hemisegments. (D) Distribution of *Ama* mRNA (in green) in the neuroectoderm/nervous system of gnathal segments at early stage 10 (left panel), early stage 11 (middle panel) and late stage 11 (right panel) by *in situ* hybridization; lower panel displays corresponding channel in monochrome. The segmental marker Engrailed (En) is shown in magenta. Yellow box indicates parasegment 2 that is determined by Scr and shows reduced expression of *Ama* mRNA. (E-H) *Ama* mRNA expression (green; lower panel monochrome) in mutant backgrounds; compare to wild type expression shown in the middle panel of (D) at early stage 11. (E) *Scr*^*17*^ mutation shows up-regulation of *Ama* mRNA expression in parasegment 2 indicated by the yellow box. (F) *Dfd*^*16*^ mutation shows down-regulation of *Ama* mRNA expression in posterior mandibular and maxillary regions (yellow box). (G) Double mutation for *lab*^*1*^ and *Dfd*^*12*^ shows down-regulation of *Ama* mRNA expression in mandibular and maxillary regions (yellow box). (H) Double mutation for *Dfd*^*16*^ and *Antp*^*7*^ shows down-regulation of *Ama* mRNA expression in the region of mandibular to thoracic segments (yellow box). (I) Distribution of Ama protein (in green) in the neuroectoderm/nervous system of gnathal segments at stage 10 (left panel), early stage 11 (middle panel) and late stage 11 (right panel) by antibody staining. Lower panel shows corresponding Ama channel in monochrome. In magenta is the segmental marker En. Red box indicates the region of NB6-4max. Orange box indicates the distribution of Ama in mandibular and more anterior regions. (J, K) Compare the Ama expression patterns to the wild type expression shown in the middle panel of (I) at stage 10. (J) *Dfd*^*16*^ mutation shows no significant change of Ama protein distribution in the region of NB6-4max (red box) presumably due to secreted Ama invading from anterior (orange box) and posterior source. (K) Double mutation for *lab*^*1*^ and *Dfd*^*12*^ shows reduced Ama distribution in the region of NB6-4max and significant reduction of Ama in more anterior regions (orange box). (L) Transheterozygous mutation for the deficiency Df(3R)BSC467 covering *Dfd* and *Ama* and *Abl*^*1*^, *Ama*^*R1*^ shows an increase in the transformation rate to 50% in NB6-4max suggesting a synergistic effect of the Dfd- and the Ama-pathway. (M) Heatshock-induced expression of *Ama* in the *lab*^*1*^/*Dfd*^*12*^ double mutant rescues the transformation phenotype of NB6-4max in 40% of all hemisegments leading to an overall transformation of 60% (*lab*^*1*^/*Dfd*^*12*^ show 100% transformation). Scale bar is 10 μm.

Since we uncovered a non-cell-autonomous function of *lab* and *Antp* in specifying the segmental fate of NB6-4max, we performed a mini-screen to identify candidate genes (Fig 4A) affected by and downstream of Hox genes. Candidate genes were selected based on existing studies on Hox downstream targets [51, 52] as well as previously identified potential modifiers of Hox genes [53–57]. We also analyzed a number of non-homeotic genes that are located in the Antp-gene cluster on chromosome 3R. Considering that Hox genes convey a non-cell-autonomous function we speculated that genes involved in signaling pathways, secreted molecules or genes known to interact with secreted molecules might be promising candidates.

These criteria supplied us with 17 candidate genes, and we tested their LoF with Eg/Repo double labeling for defects in the specification of NB6-4max identity. Whereas most of the candidates did not show any abnormal phenotype of the NB6-4 lineage (Fig 4A), the LoF of two of these genes resulted in a transformation of NB6-4max to NB6-4lab/thoracic identity: the secreted adhesion molecule Amalgam (Fig 4B, 29%, n = 160 mHs), and its potential receptor Neurotactin (Nrt, Fig 4C, 15%, n = 120 mHs)[22–25]. *Amalgam* (*Ama*) is located in the Antp-C on chromosome 3R [24]. All available *Ama* mutant stocks are combined with the mutant allele of the *Ableson tyrosine kinase Abl*^*1*^ (*Abl*^*1*^ allele alone has no effect on NB6-4max, see below). To test whether Ama is regulated by Hox genes, we analyzed the expression of *Ama* on mRNA level using *in situ* hybridization. *Ama-*mRNA is expressed from early st10 onwards with strongest expression in a repetitive pattern at early st11 (Fig 4D) in the neuroectoderm and cells of the nervous system. At late st11, *Ama* remains strongly expressed in lateral areas, while decreasing expression in the nervous system becomes restricted to the midline (Fig 4D). Throughout the neuroectoderm we observed reduced expression in the En domain until mid st11 (Fig 4D lower panels; also [24]). Surprisingly, we found that the posterior part of the maxillary (En-expressing cells) and the anterior part of the labial segment (corresponding to parasegment 2) does not express *Ama*-mRNA, perfectly matching the *Scr* expressing region (Fig 4D lower panel, yellow box). Thus, we analyzed, whether Scr might repress *Ama*, and indeed in mutants for *Scr* we could observe an up-regulation of *Ama* expression in parasegment 2, the *Scr* expression domain (Fig 4E yellow box compare to Fig 4D middle lower panel, for quantification see S6B Fig). Since we find that *Ama* is a positive regulator of NB6-4max identity, this might explain why removing *Scr* has only minor effects on NB6-4max specification (10%).

Next we tested the dependency of *Ama* expression on *lab*, *Dfd* and *Antp*. Indeed, removing the function of *labial*, *Dfd* (Fig 4F), *labial*/*Dfd* (Fig 4G) or *Dfd*/*Antp* (Fig 4H) leads to a significant reduction of *Ama* expression in the corresponding segments (compare to wild type Fig 4D, middle panel, for quantification see S6B Fig). To further validate whether *Ama* is a potential transcriptional target of Hox genes, we screened the enhancer region 3kb upstream of the coding region for the existence of conserved Hox binding motifs (described in [58–61]). We could identify Lab, Dfd, Scr and Antp binding sites that are highly conserved down to *Drosophila pseudoobscura* (25–50 mio. years distance to D. *melanogaster*, S5 Fig). To show whether Hox genes actively regulate the *Ama* transcription, we ectopically expressed *Scr*, *Dfd* and *Antp* using the *sca*Gal4 line and monitored the *Ama* mRNA expression using *in situ* hybridization (S6C Fig). Whereas *Scr* or *Antp* did not influence the wild type expression of *Ama*, *Dfd* was able to strongly upregulate *Ama* expression in ectopic positions. Taken together our analysis shows that the secreted molecule *Ama* is a transcriptional downstream target of Hox genes.

Therefore, we assume that *Ama* transcriptional regulation by Lab and Antp can non-cell-autonomously influence the segmental specification of NB6-4max. In wild type Lab, Dfd and Antp positively regulate *Ama* expression in gnathal segments, ensuring together with the cell-autonomous function of Dfd the correct specification of NB6-4max. Loss of *Ama* regulation due to loss of *lab* or *Antp* function (in single and in double mutants) appears to be compensated by Dfd function and Dfd-regulated *Ama* expression. Conversely, in *Dfd* single mutants the Lab- and Antp-dependent expression of *Ama* can rescue the loss of Dfd function in approximately 50% of all hemisegments. Finally, in *lab*/*Dfd* or *Dfd*/*Antp* double mutants lacking all sources of *Ama* induction in the area of maxillary NB6-4 leads to an increase in the transformation rate to nearly 100%. Using antibody staining for Ama, we were able to recapitulate this on the protein level (Figs 4I–4K and S6E). In the wild type CNS Ama-protein is detectable in st10 and is strongly expressed in the *lab* expression domain (Fig 4I left panel, orange box). During st11e strong Ama signal also becomes detectable in the maxillary segment (area of NB6-4max, Fig 4I middle panel, red box) whereas labial/thoracic segments start expression from mid/late st10 onwards (Fig 4I). To analyze, which cells express Ama in the nervous system and whether the receptor Nrt is expressed in NBs at the required time point we performed antibody staining of Ama and Nrt proteins and could observe Ama and Nrt proteins in both neuroectodermal cells and Neuroblasts in stage 10 (S6D Fig).

In *Dfd* single mutants Ama is still visible in the area of NB6-4max (red box) presumably due to invading Ama protein from anterior and posterior sources (Fig 4J; Lab- and Antp-dependent), whereas double mutation for *Dfd/Antp* (S6E Fig) or *lab/Dfd* (Fig 4K) lead to a complete loss of the Ama signal in the maxillary segment (red box).

A further proof for our assumption of a non-cell-autonomous component of NB6-4max specification via the secreted protein Ama would be a double mutant for *Dfd* and *Ama*. In this mutant, both pathways would be depleted. Since both genes are located in close proximity in the Antp gene cluster on chromosome 3R, we were not able to establish a double mutant fly stock. We therefore followed an alternative approach of removing *Ama* function in a heterozygous *Dfd* situation by crossing a deficiency covering *Dfd* and *Ama* (Df(3R)BSC467) to the *Abl*^*1*^,*Ama*^*R1*^ allele (Fig 4L). In this transheterozygous situation of complete LoF of *Ama* and heterozygosity for *Dfd* we could observe an increase in the transformation rate to 50% (n = 50 mHs) of all hemisegments compared to 8% in *Dfd* heterozygotes (n = 50 mHs, S6G Fig) or 29% in *Ama* single mutants (Fig 4B), reflecting the synergistic effect of both pathways. Finally, we tested whether heatshock-induced expression of *Ama* in the *lab*^*1*^/*Dfd*^*12*^ double mutant background can rescue the 100% penetrant transformation phenotype in NB6-4max. Indeed, inducing *Ama* expression at stage 9 rescued the transformation of NB6-4max in 40% of all analyzed hemisegments (Fig 4M).

Taken together, we show that the specification of segmental identity of NB6-4max depends on two pathways. First, Dfd acts cell-autonomously and removing Dfd function leads to a loss of maxillary identity and a transformation into labial/thoracic identity in 43% of all maxillary hemisegments. The remaining cases of correctly specified NB6-4max appear to be determined by a second Dfd-independent pathway, in which the secreted Ama protein, expressed under the control of *lab* and *Antp*, invades the maxillary segment from adjacent regions.

### Amalgam/Neurotactin act via Disabled regulating Abelson tyrosine kinase function to specify NB6-4max identity

Next, we wanted to understand how the non-cell-autonomous pathway of *lab* and *Antp* via Ama acts to specify the segmental identity of NB6-4max. Ama and its receptor Neurotactin (Nrt) have been identified as dominant enhancers of the Abelson tyrosine kinase (Abl) mutant phenotype in axon pathfinding [23]. Ama bound to Nrt can potentially transduce signals to the tyrosine-phosphorylated adapter protein Disabled (Dab) that genetically acts upstream to Abl [62]. Other identified factors in the Abl pathway are the antagonist Enabled (Ena)[63] and a cooperating factor named Trio [64]. We tested all factors (Dab, Abl, Ena and Trio) for a possible role in NB6-4max specification.

Ena and Trio act downstream of Abl and were identified as cytoskeleton modulators via Actin regulation [65–67] and as such could influence NB divisions per se, e.g. in changing the mode of division (asymmetric versus symmetric). In the case of NB6-4max, we could not observe a transformation phenotype in *ena* or *trio* mutants (for both n = 50 mHs; Fig 4A), and thus we assume that the cytoskeleton-associated function of Abl is not responsible for defects in NB6-4max.

**Fig 5:**
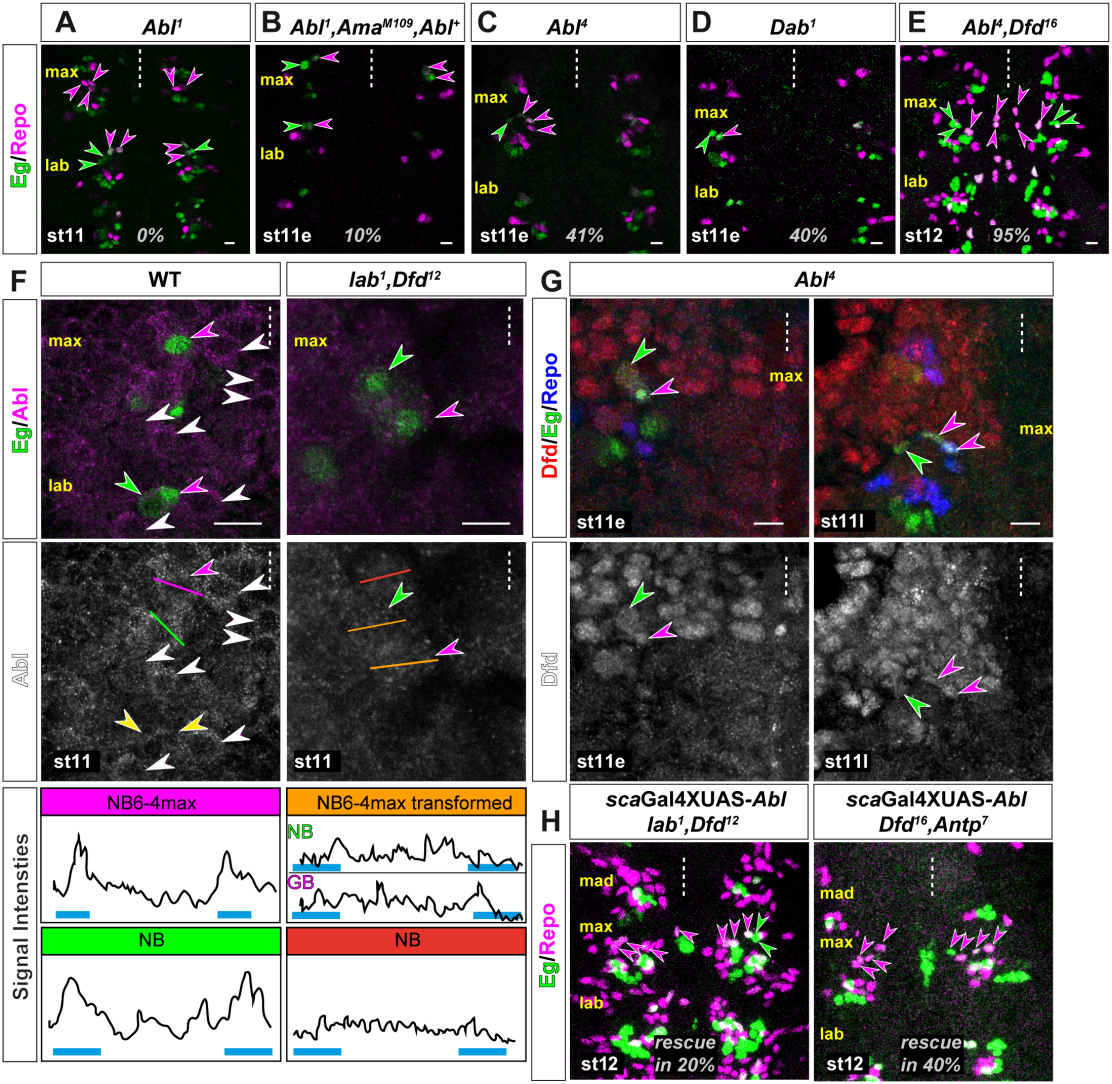
Amalgam/Nrt act via Disabled regulating Abelson tyrosine kinase function to specify NB6-4max identity. (A) Single mutation of *Abl*^*1*^ shows no transformation in NB6-4max. (B) Double mutants for *Ama*^*M109*^ and *Abl*^*1*^ with an Abl rescue construct (*Abl*^+^) show only a minor transformation rate of 10%. (C) The mutant allele *Abl*^*4*^ that has no kinase activity shows a transformation of NB6-4max in 41% of all hemisegments. (D) Mutation of the Abl interacting protein *Dab*^*1*^ shows a transformation of NB6-4max in 40% (see Fig 4A) of all hemisegments. (E) Double mutants for *Abl*^*4*^ and *Dfd*^*16*^ that interrupt both pathways show a transformation of NB6-4max in 95% of all hemisegments. (F) In the left panel: Abl (magenta) is localized in the cytoplasm with cortical enhancement in wild type NB6-4max (Eg, green; magenta arrow heads) and other NBs (white arrow heads). The labial NB6-4 has divided already (yellow arrow heads). In *lab*^*1*^ and *Dfd*^*12*^ (100% transformation of NB6-4max) double mutants (right panel) the cortical localization of Abl is disrupted. Middle panel show the Abl channel in monochrome. Lower panel show pixel intensity tracks of Abl staining across a line drawn through the cell (colors indicate corresponding line in the middle panel). In wild type (left part) NB6-4max and one other NBs as an example shows stronger pixel intensities towards the cell cortex (blue bars). This is lost in the transformed glial (GB) and neuronal precursor (NB) of NB6-4max or other NBs in the *lab*^*1*^ and *Dfd*^*12*^ double mutant situation (right part). (G) Transformed NB6-4 in maxillary segments of *Abl*^*4*^ mutant shows normal expression of Dfd (red) in early st11 (left panel) and late st11 (right panel) of lineage development. Lower panel show Dfd channel in monochrome. (H) Re-expression of *Abl* using the *sca*Gal4 line rescues the transformation phenotype of NB6-4max in 20% of all hemisegments in *lab*^*1*^*/Dfd*^*12*^ double mutants (left panel) or 40% in *Dfd*^*16*^*/Antp*^*7*^ double mutants (right panel). Scale bar is 10 μm.

Since *Ama* was found in a modifier screen of the *Abl* mutant phenotype, all available *Ama* mutants also harbor the mutant allele of *Abl*^*1*^ [23] that encodes a truncated protein with residual kinase activity [68]. Testing the *Abl*^*1*^ allele alone did not result in a transformation of NB6-4max (n = 50 mHs; Fig 5A). Moreover, comparing the double mutant stock *Abl*^*1*^, *Ama*^*M109*^ with a fly stock that includes an Abl rescue construct (*Abl*^*1*^,*Ama*^*M109*^, *Abl*^*+*^) (Fig 5B) showed the same transformation rate (both 10%; both n = 50 mHs), strengthening our finding that a) *Ama*^*M109*^ shows a transformation phenotype of NB6-4max and b) the *Abl*^*1*^*-*allele has no influence on the specification of NB6-4max. To test whether Abl kinase itself has an effect on NB6-4max specification, we analyzed the *Abl*^*4*^ mutant allele that produces a protein with catalytically inactive kinase domain [68, 69]. This allele exhibited a transformation of NB6-4max in 41% of all hemisegments (n = 400 mHs; Figs 4A and 5C). Additionally, analysis of the Abl kinase interacting protein *Disabled* (*Dab*^*1*^) mutation also showed a transformation of NB6-4max in 40% of all hemisegments (n = 60 mHs; Figs 4A and 5D).

Thus, our analysis of the Hox-mediated non-cell-autonomous pathway of NB6-4max specification shows that Ama/Nrt possibly act via Dab to regulate the Abl kinase. Intriguingly, both intracellular components Dab and Abl show a similar transformation rate like *Dfd* single mutants (all approximately 40%), again arguing for the existence of a second Dfd-independent pathway. Accordingly, a *Abl*^*4*^, *Dfd*^*16*^ double mutant indeed shows an increase in the transformation rate to 95% of all hemisegments (n = 150 mHs; Fig 5E), strongly supporting our hypotheses that a) two independent, parallel pathways ensure the proper specification of the NB6-4max identity, and b) Ama/Nrt act through regulating the Abl kinase activity.

To further prove this, we wanted to assess whether the activity change of the Abl kinase can be monitored on the protein level in WT and *lab*/*Dfd-*mutants (impairing Hox and Ama pathway), since it was shown that Abl exhibits its kinase function when localized and concentrated at the cell cortex, whereas cytoplasmic Abl acts via a kinase-independent function [70–72]. In WT embryos at st11 (Fig 5F left panels), we can observe a cytoplasmic localization of Abl that shows cortical enhancement in a lot of NBs (white arrow heads) including NB6-4max (magenta arrow head), suggesting an active Abl kinase. This cortical localization is not observable in NB6-4max in *lab*/*Dfd* double mutants in which both pathways are disrupted (Fig 5F right panels, see also tracks of pixel intensities in lower panels). On the other hand, Abl function could impinge on Dfd localization or expression. We investigated *Abl*^*4*^ mutants and we could observe nuclear Dfd expression in the glial and the neuronal precursor of transformed NB6-4max (Fig 5G). Finally, we wanted to analyze whether re-expression of Abl in the *lab*^*1*^*/Dfd*^*12*^ or *Dfd*^*16*^*/Antp*^*7*^ double mutant background could rescue the transformation phenotype. Indeed, using the *sca*Gal4 line to express *Abl* in the double mutant situation rescued the strong transformation phenotypes of NB6-4max in 20% of all hemisegments in the *lab*^*1*^*/Dfd*^*12*^ or 40% in the *Dfd*^*16*^*/Antp*^*7*^ mutants (Fig 5H).

Taken together, our analysis of the Dfd-independent pathway suggests, that Ama/Nrt ensure the proper specification of NB6-4max by positively regulating the kinase function of Abl. We conclude that nuclear located Dfd and cortical located Abl act synergistically to regulate NB6-4max identity.

Therefore, we next wanted to understand if Dfd and Abl act in parallel on the same target to ensure proper specification of NB6-4max.

### Dfd and Abl specify maxillary identity of NB6-4 lineages by repressing *CycE*

**Fig 6:**
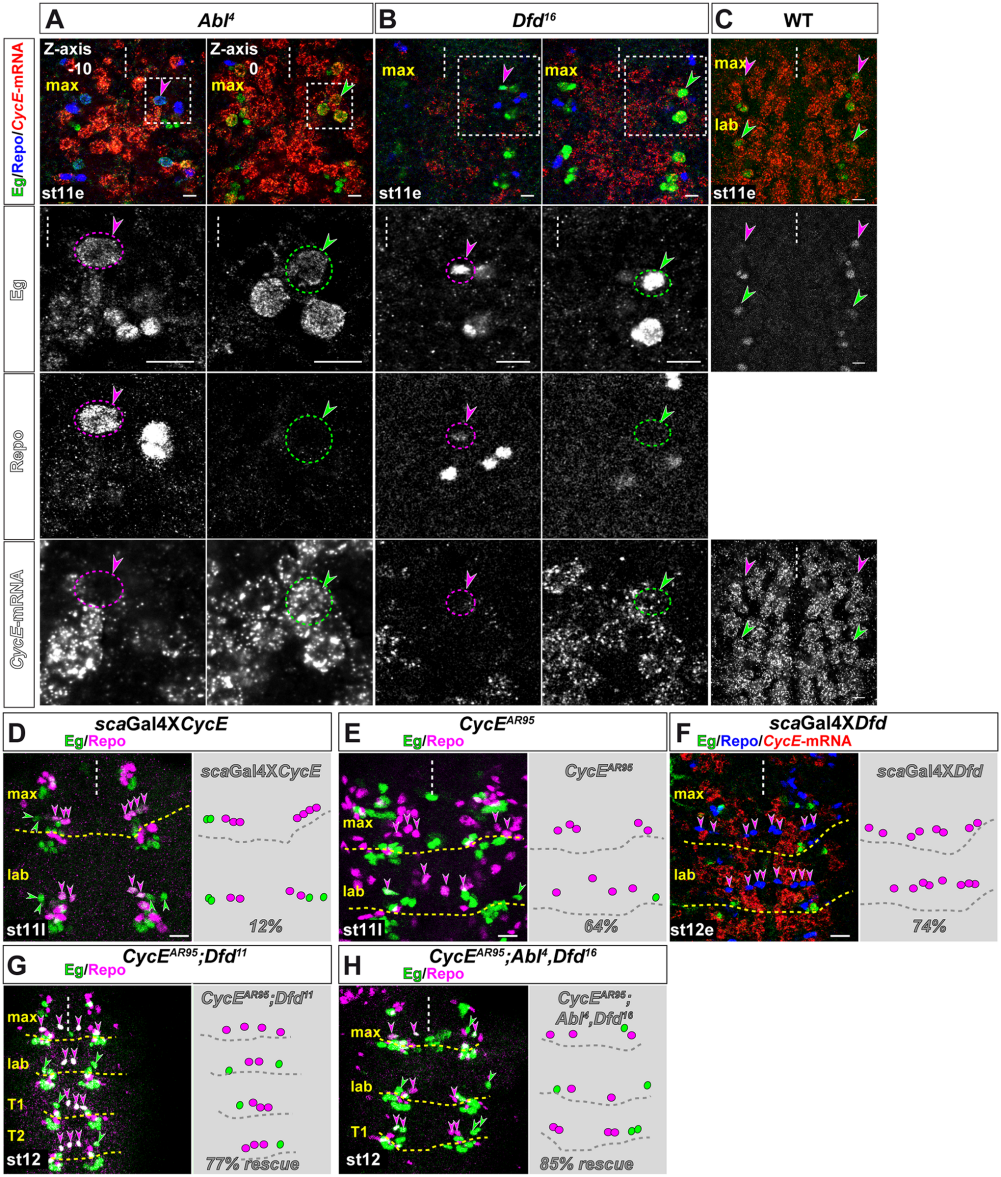
Dfd and Abl specify maxillary identity of NB6-4 lineages by repressing *CycE*. (A, B) In *Abl*^*4*^ (A) as well as in *Dfd*^*16*^ (B) mutant NB6-4max *CycE* is de-repressed and becomes distributed to the neuronal part of the transformed lineage. Whereas after the first division the glial daughter precursor (magenta circles and arrow heads, left panels) shows no *CycE* mRNA (red) the neuronal daughter precursor (green circles and arrow heads, right panels) shows clear *CycE* mRNA staining. (C) Wild type expression of *CycE* mRNA in the maxillary (magenta arrow heads) and labial NB6-4 (green arrow heads). In the maxillary segment only very weak expression of *CycE* mRNA can be found in NB6-4, whereas the labial NB6-4 shows a strong *CycE* mRNA expression. (D) Ectopic expression of *CycE* using the *scabrous*-Gal4 (*sca*Gal4) line transforms maxillary NB6-4 glioblast fate into labial/thoracic neuroglioblast fate in 12% of all hemisegments. (E) Mutation of *CycE*^*AR95*^ shows loss of the labial NB6-4 fate with only glia cells produced in 64% of all labial hemisegments. (F) Ectopic expression of *Dfd* using the *scabrous*-Gal4 (*sca*Gal4) line transforms labial/thoracic NB6-4 into a maxillary fate, generating four glial cells and no neuronal cells in 74% of all thoracic hemisegments. At this stage (st12e) no *CycE* mRNA (red) is observable in NB6-4 derived glial cells. (G) Loss of *CycE* in the *Dfd*^*11*^ single mutant leads to a rescue of the transformation phenotype of NB6-4max in 77% of all hemisegments. *CycE*^*AR95*^ mutation also leads to the loss of neuronal cells in the thoracic segments. (H) Loss of *CycE* in the *Abl*^*4*^,*Dfd*^*16*^ double mutants leads to a rescue of the transformation phenotype of NB6-4max in 85% of all hemisegments. Scale bar is 10 μm.

**Fig 7:**
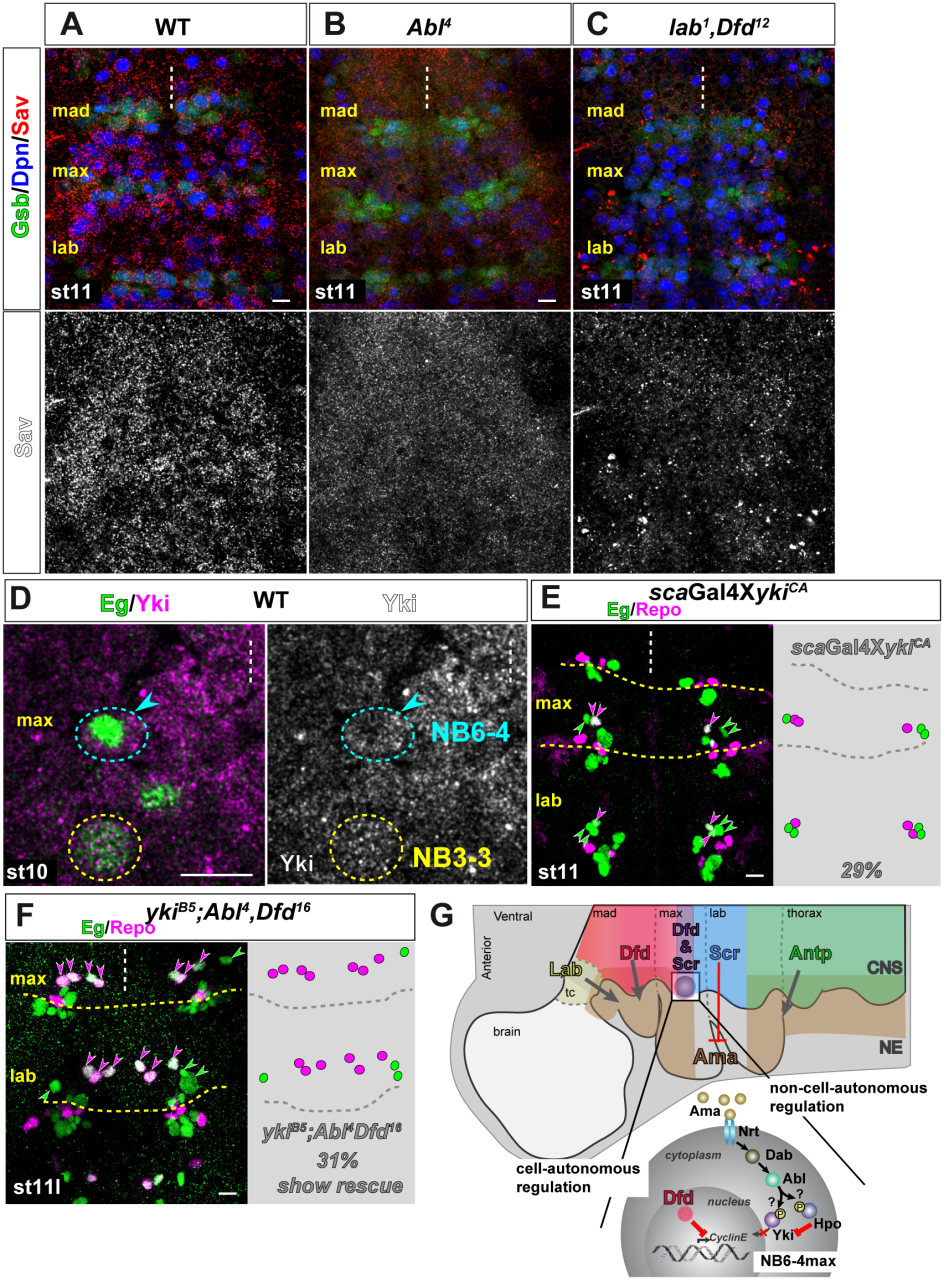
Abl potentially regulates *CycE* expression via the Hippo pathway. (A-C) In wild type neuroblasts stained with Dpn (blue) at stage 11 (A) a stable expression of Salvador (Sav, red) can be observed mirroring the active Hpo kinase. Mutation in *Abl*^*4*^ (B) or double mutation in *lab*^*1*^ and *Dfd*^*12*^ (C) show reduced Sav protein distribution, which argues for a less stable protein and an inactive Hpo kinase. Lower panel shows Sav channel in monochrome. (D) Yorkie (magenta) is localized to the cytoplasm of NB6-4max prior to its first division at stage 10. Cytoplasmically localized Yki is the inactive form of Yki. (E) Ectopic expression of a constitutive active form of *Yki*^*CA*^ using the *scabrous*-Gal4 (*sca*Gal4) line transforms NB6-4max in 29% of all maxillary hemisegments. (F) Triple mutation for *yki*^*B5*^ and *Abl*^*4*^,*Dfd*^*16*^, shows a decrease in the transformation rate from 100% in the double mutants for *Abl*^*4*^,*Dfd*^*16*^ to 69% in the triple mutants. Thus, the loss of *yki* rescues the double mutant phenotype in 31% of all hemisegments. (G) Schematic model of the cell-autonomous and non-cell-autonomous regulation of maxillary identity specification of NB6-4. Expression pattern of Antp-C genes are shown color-coded in the CNS. In the posterior part of the maxillary segment Dfd and Scr are co-expressed. Lab, Dfd and Antp positively regulate the expression of Ama (brown areas and arrows), whereas Scr represses Ama expression. Ama binds to Nrt and via Dab activates Abl that leads to the repression of CycE expression presumably through the Hippo/Salvador/Warts pathway. Whether Abl influences the Hpo kinase or Yki itself needs to be further clarified. Scale bar is 10 μm.

We have previously shown, that *abdA* and *AbdB* specify abdominal (glioblast) identity of NB6-4 by repressing the cell cycle gene *CyclinE* (*CycE)*, while CycE is expressed in thoracic NB6-4 neuroglioblasts (ground state identity) and becomes asymmetrically distributed to the neuronal daughter precursor during first division [7]. Since the loss of Dfd or Abl function leads to a transformation of NB6-4max glioblast fate into a neuroglioblast fate, we next wanted to address whether Dfd and/or Abl also modulate *CycE* expression or function. Therefore, we first studied *CycE*-mRNA expression in *Dfd*- and *Abl*-deficient NB6-4max (Fig 6A and 6B). Indeed, in transformed NB6-4max generating neurons and glial cells of *Dfd*- and *Abl*-mutants, we could observe deregulation and thus an increased expression of *CycE*-mRNA in neuronal daughter cells (Fig 6A–6C) indicating that both *Dfd* and *Abl* repress CycE expression in wild type NB6-4max. Furthermore, ectopic expression of *CycE* in NB6-4max was sufficient to generate a labial/thoracic neuroglioblast fate in 12% of all maxillary hemisegments (n = 140 mHs; Fig 6D), whereas loss of *CycE* in labial NB6-4 leads to a loss of the neuronal sub-lineage in 64% of all hemisegments (n = 100mHs; Fig 6E), similarly to *CycE* loss in thoracic NB6-4 (Fig 6G and 6H and [7]). This shows that CycE is an important target in gnathal NB6-4 that has to be regulated in order to decide whether to generate neurons or not.

Since *CycE* seems to be repressed in NB6-4max by Dfd and Abl, we wanted to test, whether Dfd or Abl can repress *CycE* in labial/thoracic NB6-4 lineages, thereby repressing the ground state identity and leading to the generation of pure glial NB6-4 lineages. Ectopic expression of *Dfd* leads to a severe loss of neuronal sublineages in labial/thoracic segments in 74% of all hemisegments (n = 100Hs) and suppression of *CycE* mRNA expression in the transformed NB6-4 lineages (Figs 3H and 6F). Moreover, in the case of labial or thoracic NB6-4 expressing ectopic *Dfd*, we could observe a perfect transformation towards maxillary fate, with 4 glial cells (instead of two in abdominal hemi-segments) and no neurons. In contrast, ectopic expression of *Abl* did not result in a repression of *CycE* in labial/thoracic NB6-4 and did not lead to a transformation towards maxillary fate. This might argue for an indirect regulation of CycE by Abl through Abl-downstream components. If Dfd and Abl act both upstream of CycE, we reasoned that CycE should be epistatic to Dfd and Abl. Therefore, we combined the *CycE*^*AR95*^ mutation with the single mutation *Dfd*^*11*^ and the double mutant of *Abl*^*4*^/*Dfd*^*16*^. Indeed, loss of *CycE* leads to a rescue of the transformation phenotype in 77% of NB6-4max in *Dfd*^*11*^ single mutants or 85% in *Abl*^*4*^/*Dfd*^*16*^ double mutants, showing that CycE is epistatic to Dfd and Abl (Fig 6G and 6H).

We conclude, that both pathways ensure the proper segmental specification of NB6-4max through repression of the cell cycle gene *CyclinE*.

### Abl seems to regulate *CycE* expression via the Hippo/Salvador/Warts pathway

Since Abl is a cytoplasmically/cortically localized kinase, we next wanted to address how Abl might repress *CycE* transcription. In addition to the higher proliferation rate of NB6-4max in Abl mutants (4–5 neurons and 3 glial cells versus 4 glial cells in the wild type), we observed a statistically significant increase in nuclear size (S7A–S7C Fig) of the Eg-positive NBs in gnathal segments. A number of *Abl* mutant embryos displayed an overproliferation phenotype with many supernumerous Eg-positive cells at the end of embryogenesis (S7D–S7F Fig). To circumvent the problem of second site mutations on the mutant chromosome, we also tested transheterozygous mutations and could observe similar results in different allelic combinations (S7A and S7B Fig). Interestingly, the same phenotype could be observed in *lab*/*Dfd* double mutants (S7G Fig), also disrupting the Ama/Abl pathway, or *Nrt*^*1*^ mutants (S7H Fig), but never in any of the single Hox mutants, in which Abl function is normal. Since this implies that Abl might regulate a pathway involved in proliferation and growth affecting CycE, and since a possible connection of the vertebrate homologue c-Abl with the Hippo/YAP pathway was recently suggested [73, 74], we wondered whether the highly conserved Hippo/Salvador/Warts (HSW) pathway might be involved in this process. At the center of the HSW pathway are the two core kinases Hippo (Hpo) and Warts (Wts) that phosphorylate and thereby inhibit the transcriptional regulator Yorkie (Yki)[75–77]. When Hpo is active it phosphorylates Salvador (Sav) that becomes stabilized [78, 79] and leads to the phosphorylation of the downstream kinases Mats and Wts. Wts finally phosphorylates Yki that upon phosphorylation retains in the cytoplasm and is inactive [80]. As soon as Hpo/Wts are inactive, Yki is no longer phosphorylated and can enter the nucleus, complex with other transcription factors like Scalloped (Sd) and start transcription of its target genes like *CycE* [80, 81].

Since so far the HSW pathway has not been implicated in embryonic NBs development, we first wanted to show the presence of the active Hpo kinase in early *Drosophila* NBs. One way to monitor Hpo activity in tissues is the Salvador (Sav) protein, since Sav is stabilized only in the presence of active Hpo via phosphorylation, whereas absence of Hpo or loss of Hpo activity leads to the destabilization of Sav and its subsequent targeting for degradation [78, 79]. We used antibody staining to visualize the levels of Sav. In wild type we can find a strong staining for Sav in nearly all embryonic NBs (Fig 7A) suggesting an active Hpo kinase. Moreover, active Hpo should lead to the cytoplasmic retention of Yki, and indeed using antibody staining, we could observe a predominant cytoplasmic localization of Yki in NB6-4max with only minor signal in the nucleus, prior to its first division (Fig 7D). Next we tested, whether Hpo activity is impaired in *Abl*-single (Fig 7B) or *lab*/*Dfd*-double mutants (Fig 7C). We were not able to monitor Yki nuclear localization in the mutant situation, but observed a clear reduction of the Sav protein (Fig 7B and 7C), suggesting that the loss of Abl function might lead to the destabilization and degradation of Sav. Since it was shown that c-Abl phosphorylates and activates vertebrate MST1 and MST2 (homologues of Hpo) and *Drosophila* Hpo [74], the loss of Sav in the *Abl* LoF situation implies an inactive Hpo kinase.

Although we were not able to show a change in the subcellular localization of Yki, we still wanted to examine the function of Yki in NB6-4max lineage development. To test if Yki is sufficient to generate neurons in NB6-4max and therefore to transform its identity, we ectopically expressed a constitutive active from of *Yki* (*YkiS168A*, *Yki*^*CA*^)[82](Fig 7E). This Yki protein can no longer be phosphorylated by Warts and as such is nuclear and active [82]. Expression of *Yki*^*CA*^ leads to a phenocopy of the *Abl*^*4*^ mutant phenotype, with NB6-4max transformed to a thoracic/labial identity in 29% (n = 60 mHs; Fig 7E) and a general increase in nuclear sizes and cell proliferation (S7I Fig). Finally, we tested the effect of *yki*^*B5*^ mutation alone or in combination with *Abl*^*4*^/*Dfd*^*16*^ or *lab*^*1*^/*Dfd*^*12*^ mutants. *Yki*^*B5*^ mutants are predominantly embryonic lethal, but a few escapers also develop into larvae. In *yki*^*B5*^ single mutants we could not observe a loss of neuronal cells in labial or thoracic segments (n = 25 mHs). Nevertheless, double or triple mutants for *yki*^*B5*^ and *Abl*^*4*^/*Dfd*^*16*^ or *yki*^*B5*^ and *lab*^*1*^/*Dfd*^*12*^ (both n = 35 mHs; Figs 7F and S7J) showed a rescue of the transformation phenotype in 31% or 34%, respectively (a drop from 100% transformation to 69% or 66%, respectively). This indicates that Yki is necessary for the generation of neurons in the transformed NB6-4max of *Abl*^*4*^/*Dfd*^*16*^ or *lab*^*1*^/*Dfd*^*12*^ mutants, and suggests that Abl kinase specifies NB6-4max identity potentially by activating the HSW pathway and thereby repressing the expression of *CycE*.

## Discussion

Along the anterior-posterior axis the CNS consists of segmental units (neuromeres) the composition of which is adapted to the functional requirements of the respective body parts. In *Drosophila* the CNS comprises 10 abdominal, three thoracic, three gnathal and four pregnathal (brain) neuromeres that are generated by stereotyped populations of neural stem cells (neuroblasts, NBs). The pattern of NBs in thoracic segments resembles the ground state while NB patterns in the other segments are derived to various degrees [14, 17]. Within each segment individual NBs are specified by positional information in the neuroectoderm [83, 84]. NBs delaminating from corresponding positions in different segments express similar sets of molecular markers [2, 14], generate similar lineages, and are called serial homologs. However, for thoracic and abdominal neuromeres it has been shown that the composition of a number of serially homologous NB-lineages shows segment-specific differences [4, 6, 85]. In the more derived gnathal and pregnathal head segments embryonic NB-lineages and the mechanisms of their segmental specification have not been analyzed so far.

### Identification of serially homologous NB-lineages exhibiting segment-specific differences in gnathal segments

Using the well-established molecular marker Eagle (Eg) which labels four embryonic NB-lineages (NB2-4, NB3-3, NB6-4, NB7-3) in all thoracic and most of the abdominal segments [20, 21] we identify serially homologous lineages of NB3-3, NB6-4 and NB7-3 in gnathal segments. The embryonic NB7-3 lineage shows segmental differences as it comprises increasing cell numbers from mandibular (2 cells), maxillary (3 cells) to labial (3–5 cells) segments, while cell numbers are decreasing from T1-T2 (4 cells), T3-A7 (3 cells) to A8 (2–3 cells)[14, 35]. Reduced cell numbers in the mandibular and maxillary NB7-3 lineages depend on Dfd and Scr function, respectively (S8A–S8E Fig). While NB7-3 appeared in all three gnathal segments, NB3-3 and NB6-4 was only found in labial and maxillary segments, and NB2-4 was not found in any of them. Our preliminary data suggest that the missing NBs are not generated in these segments, instead of being eliminated by apoptosis. For the terminal abdominal neuromeres (A9, A10) it has recently been shown that the formation of a set of NBs (including NB7-3) is inhibited by the Hox gene *Abdominal-B*[13]. Similarly, in *Dfd* mutants we occasionally observed the formation of a NB with NB6-4 characteristics in mandibular segments (10%, S8F Fig), in which it is never found in wild type.

Similar to the thoracic and abdominal segments [6] NB6-4 showed dramatic differences between maxillary and labial segments. NB6-4max produces glial cells only (like abdominal NB6-4), whereas the labial homolog produces neurons in addition to glial cells (like thoracic NB6-4). The number of glial cells produced by the glioblasts NB6-4max (4 cells) and abdominal NB6-4 (2 cells) and by the neuroglioblasts NB6-4lab (3 glia) and thoracic NB6-4 (3 glia) is segment-specific.

Thus segment-specific differences among serially homologous lineages may concern types and/or numbers of specific progeny cells and may result from differential specification of NBs and their progeny, differential proliferation and/or differential cell death of particular progeny cells. It has been shown that the segment-specific modification of serially homologous lineages is under the control of Hox genes and that during neurogenesis Hox genes act on different levels, i.e. they act in a context-specific manner at different developmental stages and in different cells (reviewed in [86]). In the thoracic/abdominal region segmental identity is conferred to NBs early in the neuroectoderm by cell-autonomous function of Hox genes of the Bithorax-Complex [8, 9, 46]. In this study we used the NB6-4 lineage to clarify mechanisms of segmental specification in the gnathal segments.

### Serially homologous NB6-4 lineages in gnathal segments are defined by cell-autonomous and non-cell-autonomous function of Antp-C genes

In segments of the trunk, the action of Hox genes strictly follows the rule of the posterior prevalence concept [12, 87]: More posterior expressed Hox genes repress anterior Hox genes and thereby determine the segmental identities. In the gnathal segments we could not observe this phenomenon on the level of the nervous system. Removing Hox genes of the Antp-C had no or only minor impact on the expression domain of other Antp-C Hox genes (see S4 Fig). Similar results were also obtained in a study that analyzed cross-regulation of Hox genes upon ectopic expression [88].

Moreover, it seems that at least in the case of the differences monitored between labial and maxillary segments Hox gene function has to be added to realize the more anterior fate. Antennapedia has no impact on NB6-4 identity in the labial segment, but specification of the maxillary NB6-4 requires the function of Deformed and Sex combs reduced. These two Hox genes are not repressed or activated by Antp (see also [88]). Also, cross-regulation between Dfd and Scr seems to be unlikely or is very weak since we observed only mild effects on the protein level and on the phenotypic penetrance. In principle Scr can repress *Dfd*, but it was suggested that this occurs only when products are in sufficient amounts [89]. In NB6-4 Dfd and Scr are co-expressed, but Scr levels appear to be insufficient to repress *Dfd*. *Dfd* seems to be the major Hox gene that cell-autonomously confers the maxillary NB6-4 fate, since the loss of *Dfd* showed the highest transformation rate and, more importantly, ectopic expression of *Dfd* in thoracic segments leads to a robust transformation towards maxillary fate. Scr does not act redundantly since in double mutants *Dfd/Scr* we did not find a synergistic effect. It might have a fine-tuning effect, as we could show that Scr influences *Ama* by repressing its transcription, whereas all other Antp-C Hox genes seem to activate *Ama*. However, since we could find only minor changes in cell identities and numbers in *Scr* LoF background, the role of Scr in NB6-4max stays enigmatic.

To our surprise cell-autonomous Hox gene function was not the only mechanism that confers segmental identity in NB6-4max. Loss of *Dfd* showed an effect in approx. 43% of all segments. Moreover, mutations of the adjacently expressed Hox genes *labial* and *Antennapedia* in combination with *Dfd* LoF showed a dramatic increase in the transformation rate of NB6-4max. We carefully studied their expression patterns on the mRNA and protein level in wild type and Hox mutant background. In no case we could find these genes to be expressed in NB6-4max or in the neuroectodermal region from which NB6-4max delaminates. This indicates that *labial* and *Antennapedia* influence NB6-4max fate in a non-cell-autonomous manner. That Hox genes can act non-cell-autonomously on stem cells was recently shown in the male germ-line, were AbdB influences centrosome orientation and the proliferation rate through regulation of the ligand Boss in the Sevenless-pathway [90]. In our study Antp-C Hox genes control the expression of the secreted molecule Amalgam, which spreads to adjacent segments and ensures segmental specification of NB6-4max in a parallel mechanism to the cell-autonomous function of Dfd. Thus, we provide first evidence for parallel non-cell-autonomous and cell-autonomous functions of Antp-C genes during neural stem cell specification in the developing CNS.

### The role of Abelson kinase in NB specification

Abelson kinase (Abl) was shown to be required for proper development of the *Drosophila* embryonic nervous system. In neurons Abl interacts with proteins like Robo [91] or Chickadee [92] and influences the actin cytoskeleton in the growth cone [93] to regulate axonogenesis and pathfinding. In this system it was also demonstrated that Ama and Nrt are dominant modifiers of the Abl phenotype [23]. In our model (Fig 7G) we propose that the interaction of secreted Ama and the membrane-bound Nrt regulates Abl function in NBs. This leads to the correct segmental specification of NB6-4max. Antp-C Hox genes *lab*, *Antp* and *Dfd* regulate the expression of *Ama* and in mutants for theses Hox genes expression of *Ama* is severely reduced, which leads to the transformation of NB6-4max due to missing Abl function and de-repression of the cell cycle gene *CyclinE*. That Abl can influence the expression of *CyclinE* was also demonstrated in a modifier-screen in the *Drosophila* eye, but the mechanism remained unclear [94]. Our genetic analysis now suggests that in NBs this might occur via the regulation of the highly conserved HSW pathway [75–77] and its downstream transcriptional co-activator Yki, which is known to regulate *CyclinE* expression [80]. The HSW pathway controls organ growth and cell proliferation in *Drosophila* and vertebrates but so far has not been implicated in embryonic NB development. We could observe Yki cytoplasmic localization in wild type NB6-4max prior to division suggesting the active Hippo pathway. Although we could not detect nuclear localization of Yki in *Abl* mutants, the loss of Yki activity in the *Abl* mutant background leads to a significant reduction in the strength of the *Abl* single mutant phenotype showing their genetic interaction and therefore supporting our proposed model in which Abl influences Yki activity. Moreover, expression of constitutive active Yki also lead to the transformation of NB6-4max and phenotypes that were similar to those observed in *Abl* mutants. We tried to assess how Abl might influence Yki activity. Work in vertebrates suggests that this could be at least on two levels: first, c-Abl was shown to directly phosphorylate and activate the vertebrate MST1 and MST2 (Hpo homologue) and the *Drosophila* Hpo on a conserved residue (Y81) [74] and second, c-Abl can also phosphorylate YAP1, which changes its function to become pro-apoptotic [73]. Our analysis suggests that in NBs Abl might regulate Hpo, since we could find changes in the stability of Salvador, which is used as a Hpo activity readout [78, 79], but we can not rule out a parallel direct regulation of Yki, since it was recently shown that other pathways like the AMPK/LKB1 pathway can directly influence Yki activity [95, 96]. Since we could observe severe over-proliferation in *Abl* or *lab*/*Dfd* mutants, that have an impaired Ama-Nrt-Abl pathway, or upon overexpression of Yki^CA^, future studies need to elucidate whether and how the proto-oncogene Abl kinase and Hox genes act on growth and proliferation or even tumor initiation through regulation of the Hippo/Salvador/Warts pathway.

## Materials and Methods

### Fly stocks

*Oregon R* (used as wild type), *lab*^*4*^, *pb*^*17*^, *Scr*^*11*^, *Scr*^*17*^, *Dfd*^*11*^, *Dfd*^*12*^, *Dfd*^*16*^, *Antp*^*25*^, *Antp*^*11*^, *Dfd*^*16*^*/Scr*^*4*^, *Dfd*^*16*^*/Antp*^*7*^, *lab*^*1*^*/Dfd*^*12*^, *Scr*^*4*^*/Antp*^*25*^, *CycE*^*AR95*^, *Abl*^*4*^, *Abl*^*1*^, *Nrt*^*1*^, *hd*^*Ff*^, *disco*^*1*^, *dpp*^*hr27*^, *ems*^*4*^, *Dab*^*1*^, *ena*^*23*^, *trio*^*8*^, *sas4*^*s2214*^, *grappa*^*61A*^, *zen*^*1*^, *bcd*^*12*^, *ftz*^*11*^, *Alh*^*r13*^, wg^8^ (all strains are balanced with *TM6b)*, *iab2-lacZ Antp-Hu e*, UAS-*CyclinE*, UAS-*n-lacZ*, UAS-*ykiS168A* (*yki*^*CA*^), *scabrous*-Gal4, *engrailed*-Gal4, Hsp70-*Ama*, UAS-*Abl* (all from Bloomington); *Ama*^*M109*^*/Abl*^*1*^, *Ama*^*M109*^*/Abl*^*1*^, *Abl*^*+*^ *Ama*^*R1*^*/Abl*^*1*^ and *Nrt*^*M54*^*/Abl*^*1*^ (gifts from E. Liebl) [23]; *tsh/tio* ^8^and *tsh* ^*2757*^ (gifts from L. Fasano) [97–99], *yki*^*B5*^ (gift from J. Knoblich, [80], *gbb*^*1*^ and *gbb*^*2*^ (gifts from K. Wharton) [100], *mspo*^*c26*^ (gift from A. Nose) [101], *wg*^*cx4*^ (gift from J. Ng) [102], UAS-*Dfd*, UAS-*Scr*, UAS-*Antp*, UAS-*lab* (gifts from A. Percival-Smith) [88, 103–105].

### In silico binding sites for Hox genes

To identify conserved Hox binding sites in the *Ama* locus we used the EvoPrinterHD (http://evoprinter.ninds.nih.gov/) and compared *Drosophila melanogaster* to *Drosophila pseudoobscura* and *Drosophila simulans*. Entry sequence was the *D*. *melanogaster Ama* gene locus plus 3kb of upstream sequence from the 5’UTR of *Flybase* release 2015_01.

### Immunochemistry

Following dechorionization in 7.5% bleach, embryos from overnight collections were devitellinized and fixed in heptane with 4% formaldehyde in 0.3% PBT buffer (1x PBS with 0.3% Triton, C. Roth) for 20 minutes. Dechorionisation and dehydration was accomplished by vortexing and 10 min wash in methanol. The heatshock of Hsp70-*Ama* in *lab*^*1*^/*Dfd*^*12*^ double mutants was induced at embryonic stage 9 for 1 hour at 37°C. Afterwards embryos were allowed to develop until stag 13 at 25°C before fixing and staining.

Primary antibodies used: mouse (m) α-Eg (1:100, gift from C.Q. Doe)[106], rabbit (rb) α-Eg (1:500) [20], rb α-Repo (1:500, gift from T. Halter) [37] and guinea pig (gp) α-Repo (1:1000, gift from B. Altenhein) [107], gp α-Dfd (1:100, gift from W. McGinnis) [108], rb α-En (1:100), rb α-Dfd (1:20, both Santa Cruz), m α-Engrailed (1:5), m α-Nrt (1:100), m α-Ena (1:1000), m α-Dab P4 (1:50), m α-Wrapper (1:20), m α-Antp (1:20), m α-Scr (1:20), m α-Prospero (1:10, all DSHB), rb α-Pb (1:50, gift from T. Kaufman) [109], rat α-Lab (1:10, gift from F. Hirth) and rb α-Lab (1:100, gift from H. Reichert), rb α-Ama (1:500, gift from I. Silman) [25], rb α-Abl (1:500, gift from E. Giniger) [62], m α-beta-gal (1:750, Promega), chicken α-beta-gal (1:1000, Cappel), rb α-Castor (1:500, gift from M. Odenwald) [110], m α-GFP (1:500, Covance), rat α-Gooseberry distal and rat α-Gooseberry proximal (1:2, gift from R. Holmgren) [111], gp α-Zfh1 (1:500), gp α-Deadpan (1:1000, both gift from J. Skeath), gp α-Runt (1:500, gift from J. Reinitz) [112], rb α-Ey (1:1000, gift from Uwe Walldorf) [34], rb α-Ind (1:3000, gift from T. von Ohlen) [113], rb α-Yki (1:200, gift from K. Irvine) [82], rb α-Sav (1:100, gift from J. Jiang) [114], rb α-pH3 (1:1000, Abcam).

The secondary antibodies used were α-mouse-Cy3, α-rabbit-FITC, α-rabbit-Cy3, α-guinea pig-Cy5, α-rat-Cy5 (1:500, all from donkey, all Jackson Immunoresearch Laboratories) and donkey α-mouse-Alexa488 (1:500, Molecular Probes).

In situ probes against *Ama*- *Dfd*-, *Scr*-, *Antp*-, *gcm-* and *CycE*-mRNA were generated by PCR using the DIG-RNA and FITC-RNA labeling kit (Roche Applied Science) from an embryonic cDNA library and genomic DNA. The probe for *labial-*mRNA was generated by R. Urbach. In situ hybridizations were performed according to standard procedures using a 40% formamide hybridization solution.

The Leica TCS SP2 and SP5 confocal microscope was used for fluorescent imaging, and images were processed using Leica Confocal software, Adobe Photoshop and Adobe Illustrator. Pixel intensities and tracks of pixel intensities were measured using the Volocity software. Statistical analysis of the nuclear size was analyzed using the sigmaBlot v.11 software.

### Measurement of Hox gene expression domains

Embryos from stage 5 to stage 9 (according to Campos-Ortega and Hartenstein 1997) where stained with Alkaline Phosphatase (AP) and *in-situ* RNA-probes for *lab*, *Dfd*, *Scr* and *Antp* mRNA. Whole-mount embryos where documented with a Zeiss Axioplan microscope using a 40x objective and pictures where digitalized with a Kontron Progress3012 camera. The measurements of the Hox gene expression domains were taken with the Adobe Photoshop CS4 measurement tool. The total length of the embryo was taken as 100% value and the extent of the expression domain calculated accordingly from the anterior pole. All single values where evaluated statistically with Microsoft Excel to attain a median for each stage and Hox gene.

## Supporting Information

S1 Fig Identification of Eg-expressing cells in the gnathal segments. (A) Triple-staining for Eg (green), Gsb (red) and Even-skipped (Eve, blue) identifies the Eve-positive progeny of NB3-3. Right panel shows Eve and Gsb staining only. White arrows indicate the position of NB3-3 cell lineage. (B) Double staining for Eg (green) and Eyeless (Ey, magenta) identifies NB7-3 cell lineage (white arrows). (C) In labial segments NB6-4 divides asymmetrically and Prospero (Pros, magenta, upper panel) and the *gcm*-mRNA (magenta, lower right panel) are unequally distributed during the first division to the glial precursor (magenta arrow head). *Gcm*-mRNA in the maxillary NB6-4 is distributed to both daughter cells during the first division (lower left panel). (D) At stage 16 many more cells express Eg compared to thoracic or abdominal segments. (E-K) Identification of midline cells based on expression of the markers (all shown in magenta) Single-minded (Sim, E) [41], Runt (F), Engrailed (En, G), Castor (Cas, H), Zinc finger homeodomain 1 (Zfh-1, I), Wrapper (J) and Pros (K). The Eg-positive midline cells (white circles) have been identified as progeny of the MNB [42]. (L-O) Identification of ventrally located clusters of Eg-positive cells (white circles) as progeny of the NB 5–3 using the marker proteins (all in red) Runt (L), Ey (M), Ladybird-early (Lbe, N) and Intermediate nervous system defective (Ind, O). Since these cells express Gsb but not En, they seem to derive from row 5 NBs (L)[32, 43]. The expression of Runt (NB5-2 and NB5-3)[29] in combination with Eyeless (NB5-3), Ladybird (NB5-3 progeny and NB5-6)[44] or Intermediate-nervous-system-defective (Ind, NB5-3)[45] indicate that these cells derive from NB5-3 (L-O). Scale bar is 10 μm.
(TIF)

S2 Fig Detailed expression analyses reveal late progeny of NB6-2, NB4-3, NB4-4 and NB5-6 in the maxillary and/or the mandibular segments as extra Eg-positive cells. (A) Identification of extra Eg-positive cells (green) in the mandibular segment at stage 14. Markers used are shown in red: Ey, Ming, Huckebein (Hkb), Empty spiracles (Ems) and muscle segment homeobox (Msh). The combination of marker expression of Ey, Ming, Hkb in addition to Ems labels NB4-4 (yellow arrow heads), and in addition to Msh labels NB4-3 (cyan arrow heads). (B) Identification of extra Eg-positive cells (green) in the maxillary segment at stage 14. Markers used are shown in red, Ey, Ming, Hkb, Lbe, Ind, Dachshund (Dac) and Msh. Expression of Ey, Msh, *ming*-lacZ, *hkb*-lacZ and Dac and missing expression of Gsb labels NB4-3 (white circle). The combination of Lbe and Gsb and missing expression of Ey labels NB5-6 (cyan circle). NB6-2 is identified by expression of Eg, Gsb and Ind (blue circle). NB5-3 Eg-positive cells (yellow circle) are identified by expression of Gsb, Lbe, Ey, *ming*-lacZ and *hkb*-lacZ. Scale bar is 10 μm.
(TIF)

S3 Fig Measurements of the expression domains of *labial*-, *Deformed*-, *Sex combs reduced*- and *Antennapedia*-mRNA at stages 5, 6, 7 and 8. For each stage and corresponding gene the measurements for several embryos are shown. The width of the expression domain is represented by a red bar for each individual embryo and its position is given in percent of the egg length. Along a representative image is given. The bottom panel shows the average expression domains of all analyzed genes per stage. Scale bar is 10 μm.
(TIF)

S4 Fig Influence of Hox genes on NB6-4max and *Hox* gene expression pattern in Hox gene mutants. (A) Ectopic expression of *Scr* using the *scabrous*Gal4 (*sca*Gal4) line does not alter labial NB6-4 segmental identity. (B) Ectopic expression of *Dfd* using the *sca*Gal4 line transforms labial NB6-4 (neuroglioblast) into a NB6-4max (glioblast) identity in 74% of all hemisegments. (C) In *labial*^*4*^ (*lab*^*4*^) mutants no transformation of NB6-4max can be observed—the lineage comprises four glial cells (magenta arrow heads) and no neuronal cells. (D) In *lab*^*1*^ and *Antennapedia*^*25*^ (*Antp*^*25*^) double mutants no transformation of NB6-4max can be observed—four glial cells (magenta arrow heads) and no neuronal cells are present. (E) In *lab*^*1*^ and *Dfd*^*12*^ double mutants NB6-4max is transformed into a mixed lineage distributing *gcm*-mRNA (magenta) to the glial sublineage, revealing a transformation on the progenitor level. (F) Double mutation of *Scr*^*4*^ and *Antp*^*25*^ leads to an increase in the transformation rate of NB6-4max (33%) compared to single *Scr*^*17*^ mutants (10%). (G) Expression pattern of Dfd, Scr, Antp and Lab (all in magenta) in the wild type nervous system at the indicated stages. Stainings are either in combination with Eg or En (green). (H) Expression of Lab, Scr, Antp (magenta, Scr also in red) or *Scr*-mRNA (red) in *Dfd*^*16*^ mutants. Scr protein is reduced, which is presumably due to a translational inhibition as we observed normal mRNA (right panel) levels. Lower panels show Scr or *Scr*-mRNA channel alone. (I) Expression of Dfd and Antp (both magenta) is not altered in *Scr*^*11*^ mutants. (J) Expression of Scr (magenta) is not altered in *Antp*^*25*^ mutants. (K) Expression of *Scr*-mRNA (red) and Antp (green) is not altered in *lab*^*1*^*Dfd*^*12*^ double mutants. (L) Expression of Lab (red) and Antp (green) is not altered in *Dfd*^*16*^*Scr*^*4*^ double mutants. (M) Expression of Dfd (magenta) is not altered in *Scr*^*17*^*Antp*^*25*^ double mutants. (N) Expression of Scr (magenta) is not altered in *lab*^*4*^ mutants. (O) Proboscipedia (red) is not expressed in NB6-4. Scale bar is 10 μm.
(TIF)

S5 Fig Hox binding sites are conserved in the *Ama* gene locus of *Drosophila melanogaster*, *D*. *simulans* and *D*. *pseudoobscura* (25–55 Million years). Conserved binding sites in all three species are shown in black capital letters.
(PDF)

S6 Fig Ama transcriptional regulation by Hox genes. (A) *Ama* mRNA expression (green; lower panel monochrome) in *Dfd*^*16*^,*Scr*^*4*^ mutant background counterstained with Engrailed (magenta). Compared to wild type expression (see middle panel in Fig 4D) *Ama* is upregulated in the Scr-expressing domain and downregulated in the Dfd-expressing domain (both marked with yellow box in lower panel) at early stage 11. (B) Quantification of the pixel intensities of *Ama*-mRNA *in situ* hybridization in the Scr- or Dfd-expression domain of wild type and different Hox mutants (see Fig 4D–4H). Signals were normalized to the pixel intensity of *Ama*-expression in segment T3 of the corresponding embryo. Loss of *Scr* leads to an increase of *Ama*-expression, also in the *Dfd/Scr* double mutation. This might explain the reduced transformation rate of NB6-4max compared to *Dfd* single mutants (see Fig 3D and 3F). Loss of *Dfd* alone or in combination with *Scr*, *lab* or *Antp* leads to a strong reduction of *Ama* expression in the Dfd-expression domain. The y-axis shows the deviation of the pixel intensity in percentage from the wild type pixel intensity. (C) *Ama*-mRNA expression upon ectopic expression of *Antp*, *Scr* or *Dfd* using the *sca*Gal4 line. Only Dfd can strongly upregulate *Ama*-expression in ectopic areas (right panel). (D) Antibody staining for Nrt (green) and Ama (red, along with pH3, which is only in mitotic cells in the nucleus) at stage 10 shows that neurectodermal cells (ne) and neuroblasts (NBs, Mira positive, blue) express both proteins. (E) In *Dfd*^*16*^/*Antp*^*7*^ double mutants Ama protein is severely reduced. (F) Expression and localization of Abl (red, or monochrome in the lower panel) in wild type (left panel), *lab*^*1*^*/Dfd*^*12*^ double mutants (middle panel) or *Abl*^*1*^*/Ama*^*R1*^ double mutants (right panel). In wild type NBs Abl localizes to the cytoplasm with cortical enhancement. This localization is lost in both double mutant backgrounds. NB6-4max and labial glial precursors are marked with yellow arrow heads, neuronal precursor with white arrow heads. (G) Transheterozygous *Dfd*^*16*^ mutants show a transformation of NB6-4max in 8% of all hemisegments. Scale bar is 10 μm.
(TIF)

S7 Fig Overproliferation phenotypes in various mutant situations of the Ama-Nrt-Abl pathway, *lab*,*Dfd* double mutants or ectopic expression of constitutive-active *yki*^*S168A*^. (A,B) In *Abl*^*1*^,*Ama*^*R1*^/*Abl*^*4*^ (A) or *Abl*^*1*^,*Nrt*^*M54*^/*Abl*^*4*^ (B) transheterozygous mutants cells with big nuclei (white arrow heads) can be observed. (C) Statistical analysis of the nuclear size of Eg-positive gnathal NBs in wild type (grey, n = 12 NBs) and *Abl*^*1*^,*Ama*^*R1*^/*Abl*^*4*^ mutants (red, n = 23). The size difference is statistically highly significant increased in the mutant (t-test analysis, p<0,001). (D-I) Loss-of-function of *Abl*^*4*^ (D), *Abl*^*1*^,*Nrt*^*M54*^ (E), *Abl*^*1*^,*Ama*^*R1*^ (F), *lab*^*1*^,*Dfd*^*12*^ (G), *Nrt*^*1*^ (H) or ectopic expression of constitutive-active *yki*^*S168A*^ using the *scabrous*-Gal4 line (*sca*Gal4XUAS-*yki*^*S168A*^; I) leads to massive overproliferation in the embryonic nervous system. (J) Triple mutation for *yki*^*B5*^ and *lab*^*1*^,*Dfd*^*12*^ shows a decrease in the transformation rate from 100% in the double mutants for *lab*^*1*^,*Dfd*^*12*^ to 66% in the triple mutants. Thus, the loss of *yki* rescues the double mutant phenotype in 34% of all hemisegments. Scale bar is 10 μm.
(TIF)

S8 Fig Influence of Hox genes on gnathal NB7-3 lineages and the formation of an ectopic mandibular NB6-4. (A-C) Expression of Hox genes in NB7-3 in the gnathal segments in WT. (A) Dfd (red) is expressed in the mandibular NB7-3 and Scr (blue) in the maxillary NB7-3. (B) Proboscipedia (magenta) is only expressed in the mandibular NB7-3 lineage. (C) The labial NB7-3 expresses Antp (magenta), like the thoracic lineages. (D) At st16 the mandibular NB7-3 lineage in *Dfd*^*16*^/*Dfd*^*11*^ transheterozygous mutants is not reduced to 2 cells like in WT. Instead, 5 to 6 cells survive until the end of embryogenesis (right and left panels of smaller pictures show magnifications of the mandibular NB7-3 clusters in different single layers to indicate all NB7-3 Eg (green) and En (red) positive cells). (E) In *Scr*^*11*^ mutants the maxillary NB7-3 lineage is not reduced to the wild type number of 3 cells, instead, up to 8 Eg (green) and En (red) cells can be observed, shown in magnified single layers on the right side. (F) Formation of a mandibular NB6-4 lineage in 10% of *Dfd*^*16*^ mutant hemisegments. NB6-4 glia cells are identified with co-expression of Eg (green) and Repo (magenta) in a possible position of an ectopically formed NB6-4 lineage. Scale bar is 10 μm.
(TIF)
