# Supplementary figures and images for: Cell-Autonomous and Non-cell-autonomous Function of Hox Genes Specify Segmental Neuroblast Identity in the Gnathal Region of the Embryonic CNS in Drosophila
Source: PLoS Genet. 2016 Mar 25;12(3):e1005961. doi: 10.1371/journal.pgen.1005961 (PMC4807829; doi:10.1371/journal.pgen.1005961)

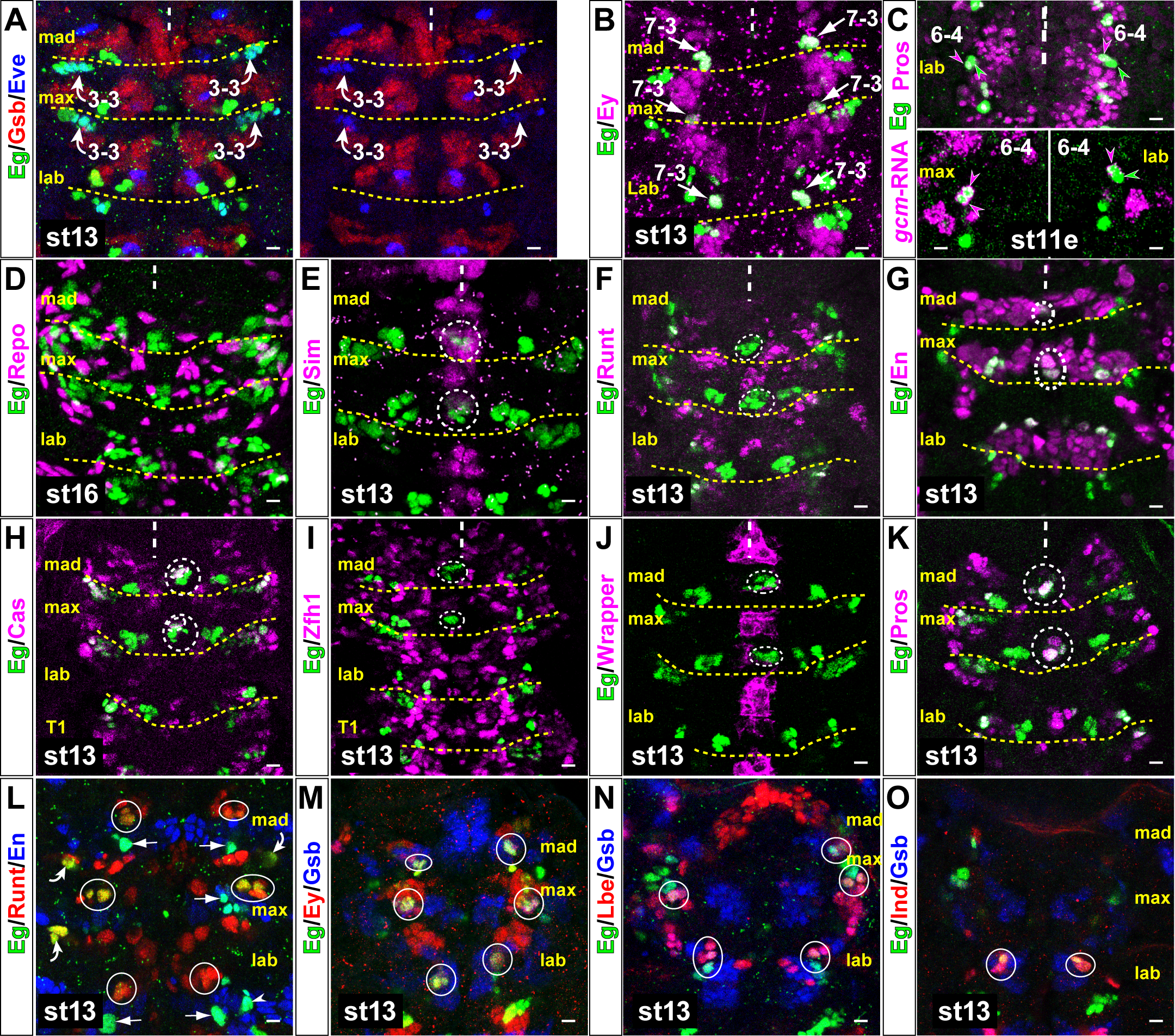

Supplement: S1 Fig — (A) Triple-staining for Eg (green), Gsb (red) and Even-skipped (Eve, blue) identifies the Eve-positive progeny of NB3-3. Right panel shows Eve and Gsb staining only. White arrows indicate the position of NB3-3 cell lineage. (B) Double staining for Eg (green) and Eyeless (Ey, magenta) identifies NB7-3 cell lineage (white arrows). (C) In labial segments NB6-4 divides asymmetrically and Prospero (Pros, magenta, upper panel) and the gcm-mRNA (magenta, lower right panel) are unequally distributed during the first division to the glial precursor (magenta arrow head). Gcm-mRNA in the maxillary NB6-4 is distributed to both daughter cells during the first division (lower left panel). (D) At stage 16 many more cells express Eg compared to thoracic or abdominal segments. (E-K) Identification of midline cells based on expression of the markers (all shown in magenta) Single-minded (Sim, E) [41], Runt (F), Engrailed (En, G), Castor (Cas, H), Zinc finger homeodomain 1 (Zfh-1, I), Wrapper (J) and Pros (K). The Eg-positive midline cells (white circles) have been identified as progeny of the MNB [42]. (L-O) Identification of ventrally located clusters of Eg-positive cells (white circles) as progeny of the NB 5–3 using the marker proteins (all in red) Runt (L), Ey (M), Ladybird-early (Lbe, N) and Intermediate nervous system defective (Ind, O). Since these cells express Gsb but not En, they seem to derive from row 5 NBs (L)[32, 43]. The expression of Runt (NB5-2 and NB5-3)[29] in combination with Eyeless (NB5-3), Ladybird (NB5-3 progeny and NB5-6)[44] or Intermediate-nervous-system-defective (Ind, NB5-3)[45] indicate that these cells derive from NB5-3 (L-O). Scale bar is 10 μm. (TIF) [file pgen.1005961.s001.tif]

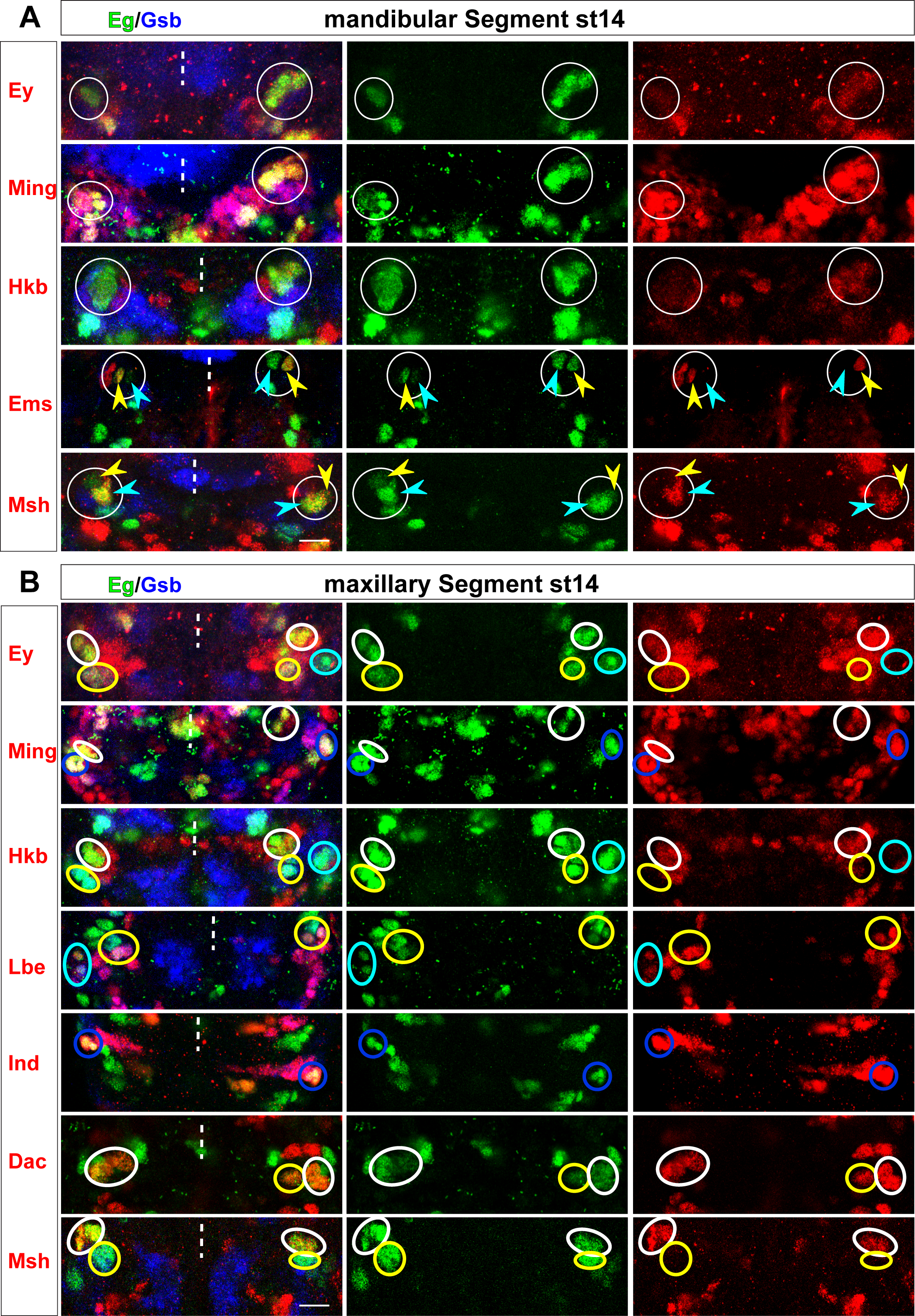

Supplement: S2 Fig — (A) Identification of extra Eg-positive cells (green) in the mandibular segment at stage 14. Markers used are shown in red: Ey, Ming, Huckebein (Hkb), Empty spiracles (Ems) and muscle segment homeobox (Msh). The combination of marker expression of Ey, Ming, Hkb in addition to Ems labels NB4-4 (yellow arrow heads), and in addition to Msh labels NB4-3 (cyan arrow heads). (B) Identification of extra Eg-positive cells (green) in the maxillary segment at stage 14. Markers used are shown in red, Ey, Ming, Hkb, Lbe, Ind, Dachshund (Dac) and Msh. Expression of Ey, Msh, ming-lacZ, hkb-lacZ and Dac and missing expression of Gsb labels NB4-3 (white circle). The combination of Lbe and Gsb and missing expression of Ey labels NB5-6 (cyan circle). NB6-2 is identified by expression of Eg, Gsb and Ind (blue circle). NB5-3 Eg-positive cells (yellow circle) are identified by expression of Gsb, Lbe, Ey, ming-lacZ and hkb-lacZ. Scale bar is 10 μm. (TIF) [file pgen.1005961.s002.tif]

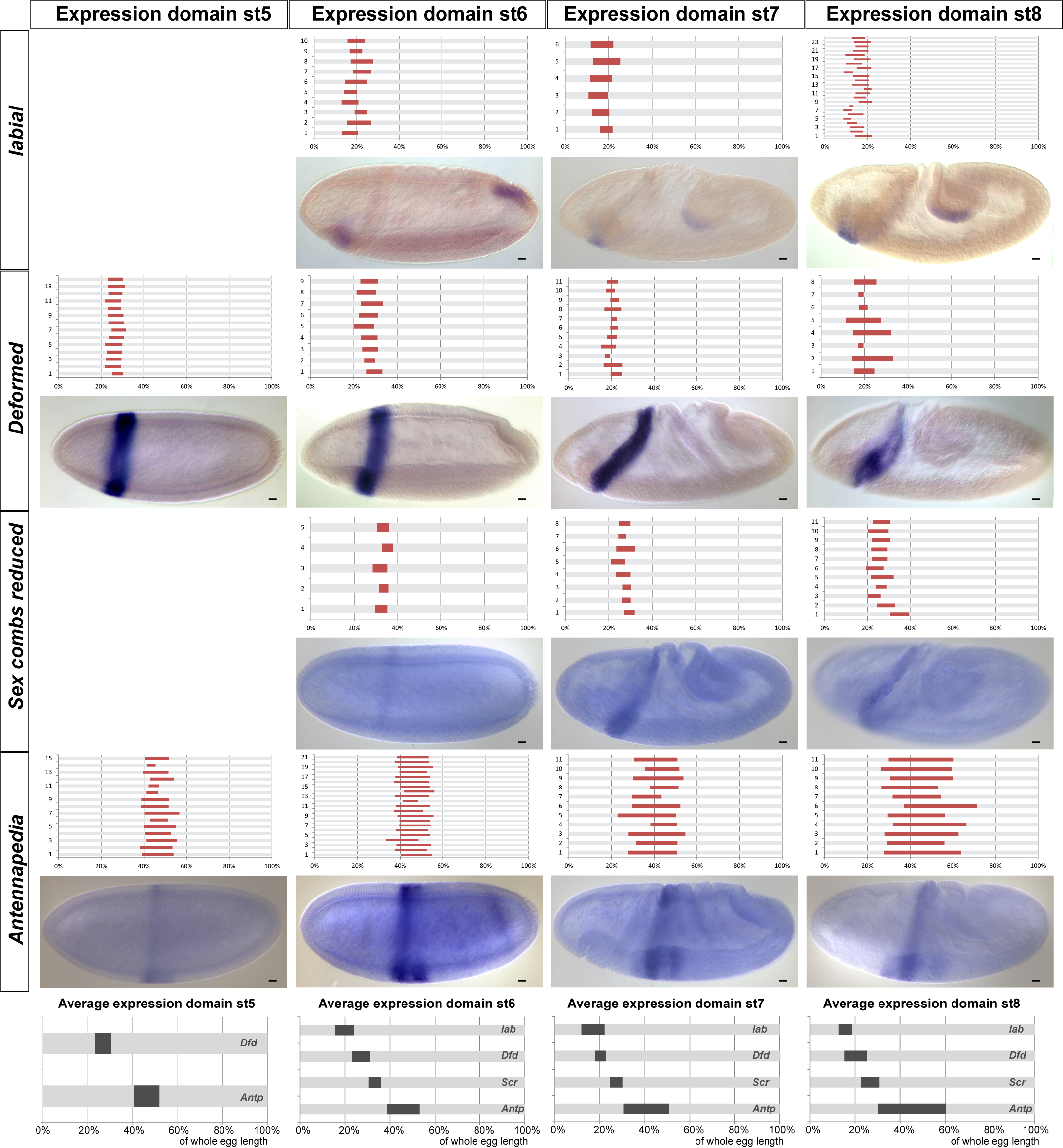

Supplement: S3 Fig — For each stage and corresponding gene the measurements for several embryos are shown. The width of the expression domain is represented by a red bar for each individual embryo and its position is given in percent of the egg length. Along a representative image is given. The bottom panel shows the average expression domains of all analyzed genes per stage. Scale bar is 10 μm. (TIF) [file pgen.1005961.s003.tif]

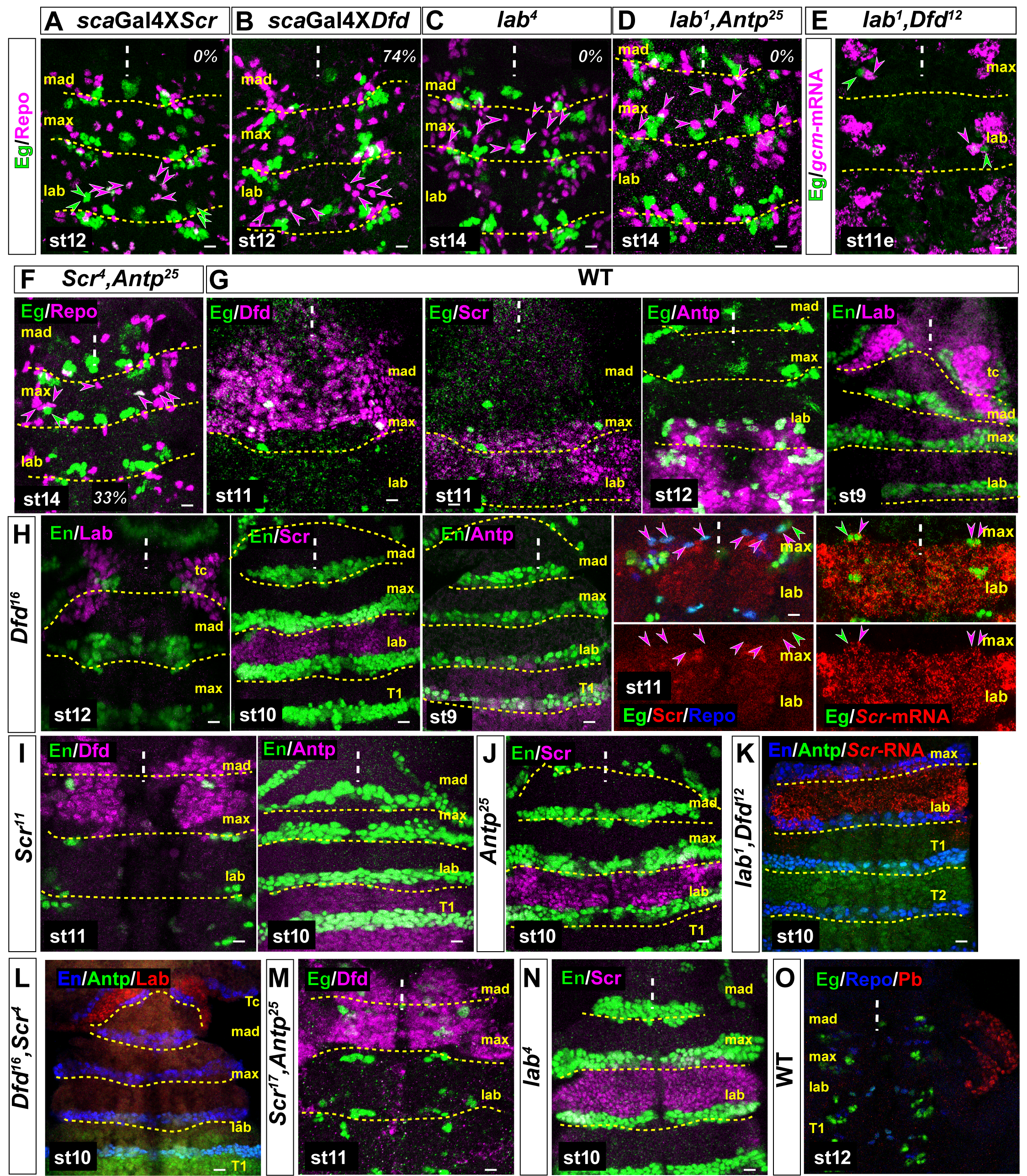

Supplement: S4 Fig — (A) Ectopic expression of Scr using the scabrousGal4 (scaGal4) line does not alter labial NB6-4 segmental identity. (B) Ectopic expression of Dfd using the scaGal4 line transforms labial NB6-4 (neuroglioblast) into a NB6-4max (glioblast) identity in 74% of all hemisegments. (C) In labial4 (lab4) mutants no transformation of NB6-4max can be observed—the lineage comprises four glial cells (magenta arrow heads) and no neuronal cells. (D) In lab1 and Antennapedia25 (Antp25) double mutants no transformation of NB6-4max can be observed—four glial cells (magenta arrow heads) and no neuronal cells are present. (E) In lab1 and Dfd12 double mutants NB6-4max is transformed into a mixed lineage distributing gcm-mRNA (magenta) to the glial sublineage, revealing a transformation on the progenitor level. (F) Double mutation of Scr4 and Antp25 leads to an increase in the transformation rate of NB6-4max (33%) compared to single Scr17 mutants (10%). (G) Expression pattern of Dfd, Scr, Antp and Lab (all in magenta) in the wild type nervous system at the indicated stages. Stainings are either in combination with Eg or En (green). (H) Expression of Lab, Scr, Antp (magenta, Scr also in red) or Scr-mRNA (red) in Dfd16 mutants. Scr protein is reduced, which is presumably due to a translational inhibition as we observed normal mRNA (right panel) levels. Lower panels show Scr or Scr-mRNA channel alone. (I) Expression of Dfd and Antp (both magenta) is not altered in Scr11 mutants. (J) Expression of Scr (magenta) is not altered in Antp25 mutants. (K) Expression of Scr-mRNA (red) and Antp (green) is not altered in lab1Dfd12 double mutants. (L) Expression of Lab (red) and Antp (green) is not altered in Dfd16Scr4 double mutants. (M) Expression of Dfd (magenta) is not altered in Scr17Antp25 double mutants. (N) Expression of Scr (magenta) is not altered in lab4 mutants. (O) Proboscipedia (red) is not expressed in NB6-4. Scale bar is 10 μm. (TIF) [file pgen.1005961.s004.tif]

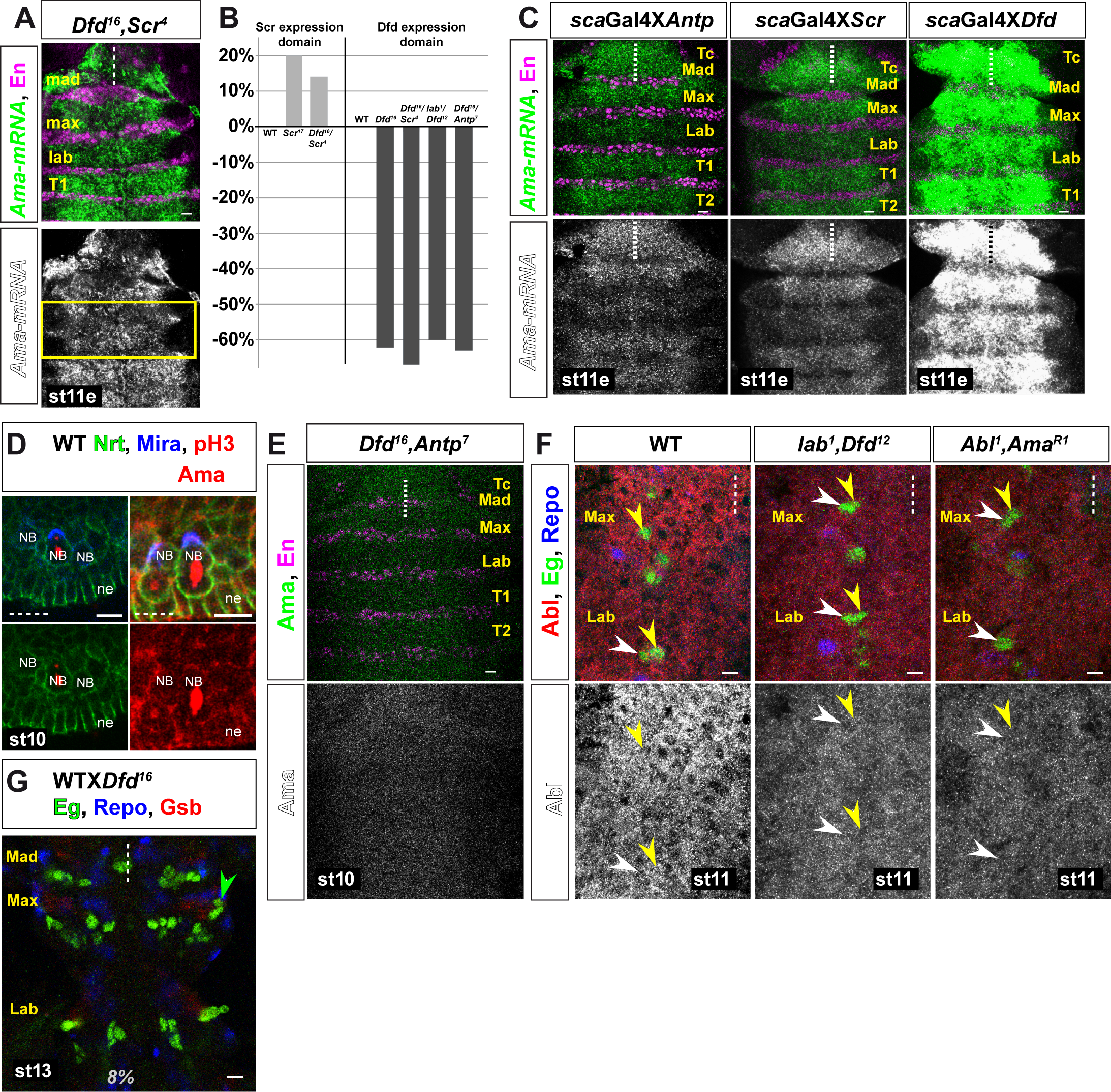

Supplement: S6 Fig — (A) Ama mRNA expression (green; lower panel monochrome) in Dfd16,Scr4 mutant background counterstained with Engrailed (magenta). Compared to wild type expression (see middle panel in Fig 4D) Ama is upregulated in the Scr-expressing domain and downregulated in the Dfd-expressing domain (both marked with yellow box in lower panel) at early stage 11. (B) Quantification of the pixel intensities of Ama-mRNA in situ hybridization in the Scr- or Dfd-expression domain of wild type and different Hox mutants (see Fig 4D–4H). Signals were normalized to the pixel intensity of Ama-expression in segment T3 of the corresponding embryo. Loss of Scr leads to an increase of Ama-expression, also in the Dfd/Scr double mutation. This might explain the reduced transformation rate of NB6-4max compared to Dfd single mutants (see Fig 3D and 3F). Loss of Dfd alone or in combination with Scr, lab or Antp leads to a strong reduction of Ama expression in the Dfd-expression domain. The y-axis shows the deviation of the pixel intensity in percentage from the wild type pixel intensity. (C) Ama-mRNA expression upon ectopic expression of Antp, Scr or Dfd using the scaGal4 line. Only Dfd can strongly upregulate Ama-expression in ectopic areas (right panel). (D) Antibody staining for Nrt (green) and Ama (red, along with pH3, which is only in mitotic cells in the nucleus) at stage 10 shows that neurectodermal cells (ne) and neuroblasts (NBs, Mira positive, blue) express both proteins. (E) In Dfd16/Antp7 double mutants Ama protein is severely reduced. (F) Expression and localization of Abl (red, or monochrome in the lower panel) in wild type (left panel), lab1/Dfd12 double mutants (middle panel) or Abl1/AmaR1 double mutants (right panel). In wild type NBs Abl localizes to the cytoplasm with cortical enhancement. This localization is lost in both double mutant backgrounds. NB6-4max and labial glial precursors are marked with yellow arrow heads, neuronal precursor with white arrow heads. (G) Transheterozy [file pgen.1005961.s006.tif]

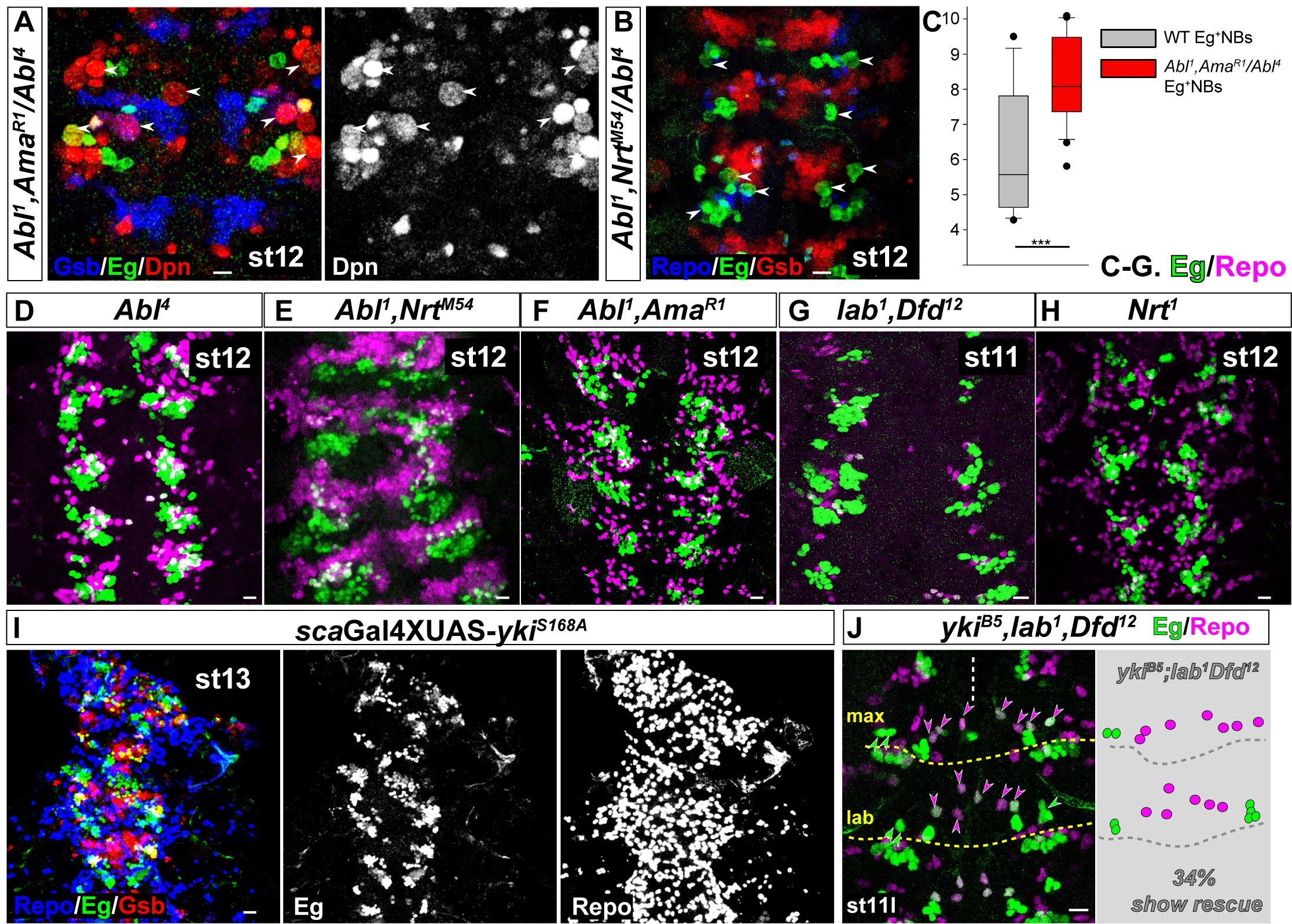

Supplement: S7 Fig — (A,B) In Abl1,AmaR1/Abl4 (A) or Abl1,NrtM54/Abl4 (B) transheterozygous mutants cells with big nuclei (white arrow heads) can be observed. (C) Statistical analysis of the nuclear size of Eg-positive gnathal NBs in wild type (grey, n = 12 NBs) and Abl1,AmaR1/Abl4 mutants (red, n = 23). The size difference is statistically highly significant increased in the mutant (t-test analysis, p<0,001). (D-I) Loss-of-function of Abl4 (D), Abl1,NrtM54 (E), Abl1,AmaR1 (F), lab1,Dfd12 (G), Nrt1 (H) or ectopic expression of constitutive-active ykiS168A using the scabrous-Gal4 line (scaGal4XUAS-ykiS168A; I) leads to massive overproliferation in the embryonic nervous system. (J) Triple mutation for ykiB5 and lab1,Dfd12 shows a decrease in the transformation rate from 100% in the double mutants for lab1,Dfd12 to 66% in the triple mutants. Thus, the loss of yki rescues the double mutant phenotype in 34% of all hemisegments. Scale bar is 10 μm. (TIF) [file pgen.1005961.s007.tif]

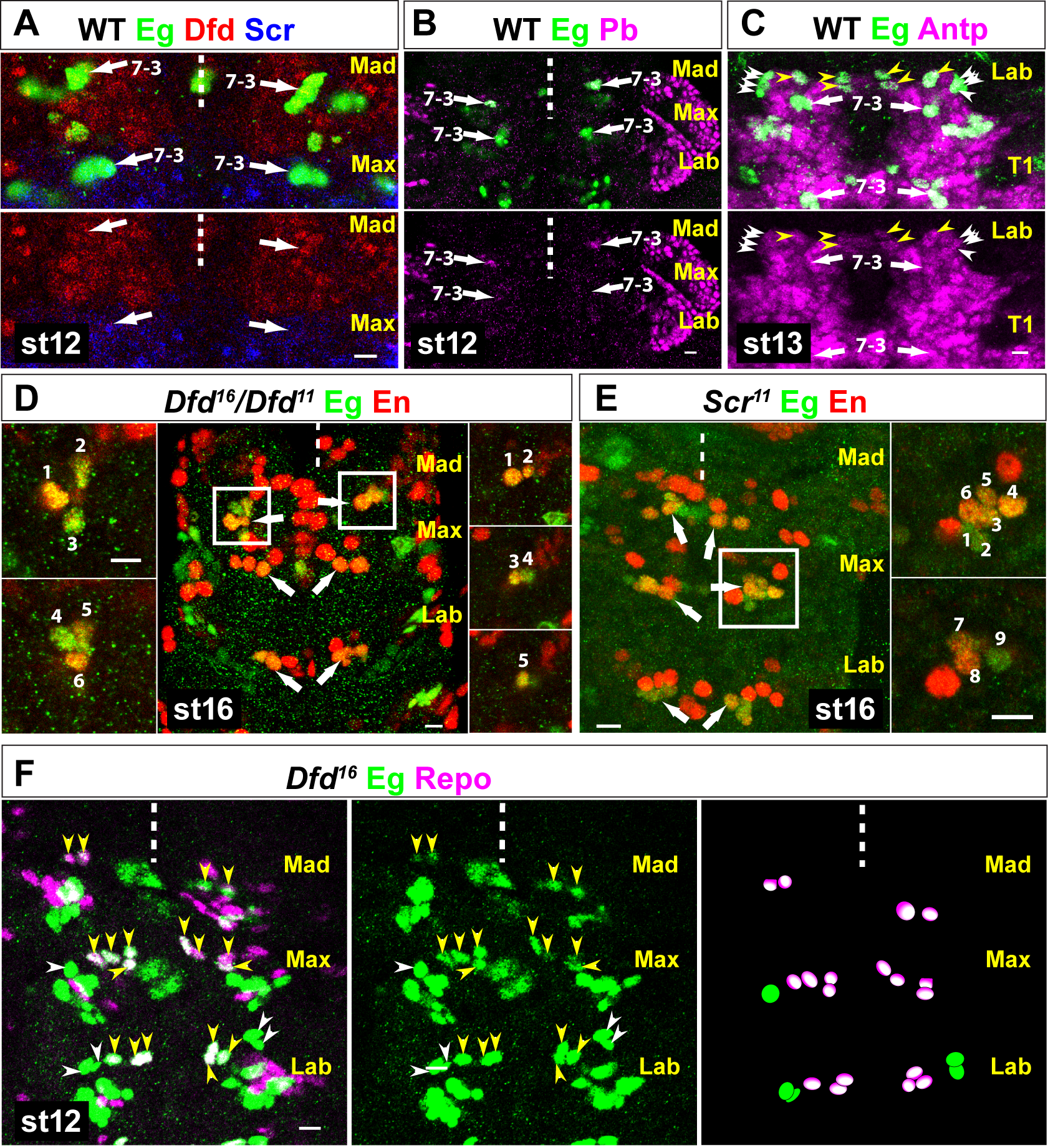

Supplement: S8 Fig — (A-C) Expression of Hox genes in NB7-3 in the gnathal segments in WT. (A) Dfd (red) is expressed in the mandibular NB7-3 and Scr (blue) in the maxillary NB7-3. (B) Proboscipedia (magenta) is only expressed in the mandibular NB7-3 lineage. (C) The labial NB7-3 expresses Antp (magenta), like the thoracic lineages. (D) At st16 the mandibular NB7-3 lineage in Dfd16/Dfd11 transheterozygous mutants is not reduced to 2 cells like in WT. Instead, 5 to 6 cells survive until the end of embryogenesis (right and left panels of smaller pictures show magnifications of the mandibular NB7-3 clusters in different single layers to indicate all NB7-3 Eg (green) and En (red) positive cells). (E) In Scr11 mutants the maxillary NB7-3 lineage is not reduced to the wild type number of 3 cells, instead, up to 8 Eg (green) and En (red) cells can be observed, shown in magnified single layers on the right side. (F) Formation of a mandibular NB6-4 lineage in 10% of Dfd16 mutant hemisegments. NB6-4 glia cells are identified with co-expression of Eg (green) and Repo (magenta) in a possible position of an ectopically formed NB6-4 lineage. Scale bar is 10 μm. (TIF) [file pgen.1005961.s008.tif]
