# Supplementary material for: Cell-Autonomous and Non-cell-autonomous Function of Hox Genes Specify Segmental Neuroblast Identity in the Gnathal Region of the Embryonic CNS in Drosophila
Source: PLoS Genet. 2016 Mar 25;12(3):e1005961. doi: 10.1371/journal.pgen.1005961 (PMC4807829; doi:10.1371/journal.pgen.1005961)
Supplement: S5 Fig — Conserved binding sites in all three species are shown in black capital letters. (PDF) [file pgen.1005961.s005.pdf]

D.melanogaster Ama + 3kb upstream - Genomic Relaxed *EvoPrint*

Black capital letters represent bases conserved in all species and colored bases represent sequences present in all species except *D.simulans* *or* *D.pseudoobscura*. Yellow labeled letters represent coding sequence of Amalgam.

Hox binding sites described in Affolter *et al.*, 1990, Ebner *et al.*, 2005, Joshi *et al.*, 2007 and Joshi *et al.*, 2010.

xxx (green)	binding site for **Exd/Dfd**	GATTAATGG (TG) (TA)
xxx (cyan)	binding site for **Exd/Lab**	TGATGGATGG<br>TGATGCATTG<br>TGATCAATTA
xxx (underlined)	binding site for **Scr**	ATTAATG (similar to Dfd)
xxx (grey)	binding site for **Antp**	CATTAGAG<br>CAATTACA

GCGAAGAGATGTAGAGATGgCACAATTGGCGGCCcTcCCTCGATGTtCaTTCTTTTGAGTAATTTaAAAATCAAT 75
AAgCGTCTAcaATAcATTTtAGAcAaATatttggacaTTTTATTTTtAAATGTTtGCCTCTaATTTAAAAGTTtt 150
TAaTCGAACAGTACGGCAaaGCTGCaAaCcGatTgAGTgCAcAGcGTtTTCaCCAGgGGGCGtgaagAgAcAGCA 225
gTagaGAGCCCCCTGTCGGGcTGtATTGaAAGGTTGTGCTCaCTGACaAAAGGTGAAtGTTCTAAaacGTCGGaG 300
GTTTATTTATTtGtAGAgATTTGCATTTGAGAaTAATAATAAAaaAAAGCTTGaATATTgTAACAAaTAAGTAgA 375
aCCAATTAAAcTTaaactCTTTGctCTGtTtGGAACTTCATAATAtTaACAttTAAttAAcAAAcAGGCAcCcCA 450
AATtTAGGAATcAATTagacAAGTgTCGAATGTTTAATTTgaATTTTTgTTAATATtATAATATTAATATGCTcC 525
AGTCcagtccagCCTGAATTGAACAAAATGTGCACTTTGTAGTTTATTcTGTTACATACTTcCGCTATTaataac 600
tacattattcaatcttaGTATCGCgagatatataaggacttGGGACAAAGAATAAGGATGgTGGAGaTCgAGTTT 675
GTTTTtCCAAatgATCTCaAACGgCTAcgTACGAAAGAAAATATCATATAATCGTTttGTAAgAcAGCaTATAGT 750
gcttgctatatgTACCTGttaGGCAGTGcATTATAAAATgTATtagAAgAATAAaAGGTAAcCAGTTGTCGAGTT 825
AaTTAgCTgtAGCGTTCgATTCTTTTGttaATTTTTtttaagttgttctactcatttcTGATgTTAAGCTAGGAT 900
TGGCaATTATTAttTTcAGGCTCcATATTTGGAAAATTCGATATGTTTAGCGTtgAAGAATATTGTgtaaatatc 975
tACAAgCATTAAGTTGCTCAGTGCATTGGCCcAAGCTTTAAcATTTTAAAACACACCCAACGAGCCAGTTGACCT 1050
TTAAGCAAGTGTCgCAAGGCCTTAAGTGAAATATTTTTTAATGACATTTgCCaAATAATTGTCGTGTTTGCTTTT 1125
GGGATCTGGCAGGAGCTTGTTGCTGTTGGCCGGGCTTTGCTGCTTCTGCCGCTGCCAACTGGCAACAAAAGTCAA 1200
TTCACAGACACTCCAGTCGACGGAGTCGGAGTGCAGCCAAAAGGCAAACAAAATACGtTCGCATAGGCAAAGCCC 1275
AAGGATACACACAAAGGTACAGTGGTTgCTCTGACTACAAATACAAATTAAACAGGgccagagctttaataccaa 1350
Ttcgataccattgcaacaacagcatttcaaatgctactttttatatcagataaagataggtagaaaaggctata 1425
Tttacgttaaaggcaagctgatattttagaaatttattcaaaaacaaaattaattactttgacacataatattgt 1500
atttagtcatctgactagaaactagaaatctctgaatgtaacgaagttctgtgcattcttacAAATaTTAcAAAA 1575
AGTATCtgctagaTACCACTGTGCGGAACTGGGCCACAAATTCAgACGCAGTGTGTGACAAAGGACTTCTCCCTT 1650
CCCCGCTCGGCGACCCTGAATGCTGAATGCCCCCGTGTGTGCCGCTGCTGAAGCGAAACACCCAAAGAGTCACTG 1725
ACAGAAACAGGATTCGGGTAATAAATTACAAAATTTGACATTTTCTGTTTTGGATCCGCTTGCGCCTGAATGCGA 1800
GCGGCAGCCCTCTCTGTCGCACGCCGTCTAGCCGTCTCTTTCGTTCGGTTTCGGTGTGTGTCCAGTTCGGTCGGG 1875
CCAAGTCGAGCGTTGCATGAAGCAGCCGGACCTGCCAGGACCTGGACCAGGACCTGAACCTGCATCCAAgAGGCA 1950
TCGCCGCAATGGTCATAATGACGCTGCCGGGATGCAGCTGAGGaCAAGGAGAGCCGtCaGTGGgcAGCGaGAAAG 2025
GGACGGAATAGAAGCGCCCTATGCGGTCCGttGATAGTTAACTGGAAATTTATTATTTTACACAATTTACAATTA 2100
TCTGATTCGAATGTGCACTCAGcGACGAGGATTCCGGCAGATGGGcAGTACGAGTGAGAGTGCGGGggTCCaAGt 2175
TCGGACGACAGATGCAGTTGtcCTCCCATGGGAACGGCCTCATTTCAGCAGAAGTCACCGAAACGATTTAAGATG 2250
TGATTTAATTTTCCAGAAACTGTGGCCATAGTTTCGTCGCTCTTCGGGGGAATTTCTTCGCCAGACAGACAGCGT 2325
CGTCATCAATCATCTcGCAGAGTAAAGCCGGGTTAACCTCGATGGGTTTTAGGGGGAaACTTGGCCCGGGGCTGA 2400
TCAGGAaTCaGGCTGGAAATATGAAAATTAAATGCGTTTGGGCaGCAGAAAGTTTTCGAATGGTTCGGTGCTTAT 2475
ACAACAAGTGAAaTTTtgCAcATcAAaCGgccTTTGACCACaTTTAATCcTTAAATCAtCTgCCATAGgAACTAA 2550
TTGATCGAGATCCAGACACaTATtAAAcacATcAATATAtATacacaAATATAATCAAtATAgaCTATGTTATAT 2625
TTcgacgtttcctatcctcgtatgctgtGAGTACACATATTAACCtTCAGCCGTgTAAAGAAATTTCTAcGAACA 2700
GCCAAGTTAAAGGTacCCAAATTTCCCGAATaCTGCTCTTTAAATATCCTTCAGAATTTCCCAGCATGGCTGAAC 2775
CAGGACTCGATTCAAGTtCCACCCAATGCCTTGTaCACCACCCCgTTGTTTTTGCCTTTTCGCGCATGCGCCCCT 2850
GCTCCGGCGCAAAAGAGACAGCACTAAtGAGACGGCGCTGGGATCGCTGGCATTTCCAGAGCGGctAGGATATTC 2925
CAGGGACCGCTCGTaaTCGTTGCTGTTGTTGTTAGCCGCCAGCGGAAAGTAGtGCGCCATGCCATAAATCACACA 3000
AATGGGTAGAGTTCCAACTTTCCCACCCcGCCGCtGCTCCTTTTaTTGTCCTTCCACGAACCCCGAAACGTTTAT 3075
GGCCAAATGGCGGCACgAATTTGAATTTGAATTTCGCACGCGGTCTCTCAGTTCGCCTGCGGCCGCTTTCGCTGG 3150
CGCTTTGTTTTTGCAACTGACCGCTTATCTTGGGGCGGGGGCTGTCAAGTGGGcgGGCAGGaGGGCCAcAGGCA 3225
GAGGCAtAAACaACAGCAGCGAGCAGCAAAACAAATCAATTAAAGACCAGAAGGAAAGTGCATTGATAACTGAGC 3300
CCGAAGTCAAACTCACGCACAAGTAAACAATTTAAATACGAaCTaAAATGCAAACACAGATACAAAATACAGATA 3375
GAGATACTGAACAAACACAGATACAAATACTGTGAAGGCGACAAtGTACATAACCGAGGGGTGATAAGATCCAAA 3450
ACGTCGATGACTTCTTCATCGGCAAATACACTGAAAAAAGCTGtTACTaATTATTAAaAAATATGcAAtATAATA 3525
AAAGGTAAAcTATTTTttttactaaattttaaactatttactaaacactgcCATTTAAAATCTTTTTTCACATCc 3600
TTAACTACAaAAAAATtAAtTTTAAATCAAATTGATttCGTTTACCTGgCTATTCAtTaaaAAAAAAAAtAAGTA 3675
tTGATATATCgGAAAACtAAGTTATCTccgTTTTTaccAGTGTAtCcATTAaGAAGCAACGTTGTCTAGACACAG 3750
CCGCGGTAATCAATCATAATGgTGTCTATCTGCtTCATTCTCGCGGTGTACAAATTTCGGGACTTTCCTTGAGAA 3825
ACCGAGCCaTCGACGTCGGCGCGTCAGGAAGAATACaAAAAtAAGAACcCGATTCTCAGAaCCTCAGCAGAGATA 3900

CGGATTTTTGAGCCGAGCTCGGTCGCCTCTTTCAATTCAGTCGAGTCCCGAACGCGAAGTGAAGCGCATTGAACC 3975

CACAttgaactgaGAGATAGTCGAGGAAAAAGAGTCACAAAATCAACAGCAACATCCTGTAAATAAACAGAAAtG 4050

TATGAATTATATTAAAATAAGACGATAAAgCTGAaAAGTGTGTaCaAGCcTTATAAATGTTTCAAAACGAGTGTG 4125

TGTGCAGAATTCTTTCcttcttatttacttttgaaagatactttTAAAGATATACTTGTGAcCTTtAAATTATTA 4200

TTCTATAaTTGAACcTAGTTTTAGTTTATTATATTGAATTCGAAGATGGGTtAAAaatgGCAGTTCTCCATTACA 4275

TACGAAATATTTAGAATGGCTAAcGCGATCAAAAGACTCAACATAAAGTGATACACTaaTAAATCTCAAATCTAT 4350

TTTGATTTCTTACAGACCAGACATGGCGCGTCTGCGGCTTCtaATCGGTCTTATAtTCTGCCTTGCGATCAGCtT 4425

GGAtTCGGTGCTTaGTGCCCCAGTGATCAGCCAGATCAGCAAGGACGTGGTGGCCAGTGTGGGCGACTCTGTGGA 4500

GTTCAACTGCACCGTGGAGGAGGTGGGACAGCTCTCGGTGAGCTGGGCCAAGCGTCCCAGCGAGTCGGACACCAA 4575

CTCGGTGGTGCTCTCCATGCGGAATATCCTCAGTCTGCCCGACCAGCGGTACAACGTAACGGTCACCGAAGGACC 4650

CAAGACCGGAAGTGCCATTTACACCTTCCGCATTCAGAACATCGAAGTCAGTGACATGGGTCCGTACGAGTGtCA 4725

GGTGCTtGTTTCGGCCACGGAGAAGGTGACCAAGAAGCTGAGCCTGCAGATCAAGACCCCGCCGGTGATTGCGGA 4800

GAACACCCCCAAGAGCACCCTGGTGACaGAGGGACAGAATCTGGAGCTGACCTGCCATGCCAATGGCTTTCCCAA 4875

GCCtACGATCTCCTGGGCCCGCGAGCATAATGCaGTGATGCCtGCCGGtGGCCATTTGCTGGCCGAACCAACTCT 4950

CCGGATTCGTtCCGTGCACCGgATGGATCGCGGCGGCTACTACTGCATCGCGCAGAACGGAGAGGGCCAGCCGGA 5025

CAAGAGGTTGATCCGGGTCGAaGTGGAGTTCCGTCCGCAGATTGCCGTCCAGAGGCCCAAGATCGCCCAaATGGT 5100

GTCCCAtTCCGCGGAGCTGGAGTGCTCCGTGCAGGGCTACCCGGCTCCGACTGTAGTTTGGCACAAGAATGGCGT 5175

GCCCCTGCAGTCTAGCCGGCACCACGAGGTGGCCAACACCGCCTCCTCCTCGGGAACCACAACCTCTGTGCTGCG 5250

CATCGAtAGCGTGgGCGAGGAGGACTTTGGGGACTACTACTGCAATGCCACCAATAAGCTGGGACACGCCGATGC 5325

GCGACTGCATCTCTTCCAGACCGTCATCCCAGTGCCCTCCTTGTCGTAATAAGCCCTCAACGTCTGATTTCCATT 5400

CCCACGCAACAAGACTGGAACCCTCACTTTCtCCTCTCGtCTCGCTTTTTCGGCAATTCGCCAGTCCGGATGCGG 5475

tCTGTGTTTATATTATTAATGATTCATTCAATTAGCTCCCATTCAGACTTCATTATTTTCGGACACACTTGAGAT 5550

CGAGCCACCGCTTGTGCCTTATGAATTTTGCTCTGCATCCGTCGGCTtGTGCAAACACACATTAATATGCTTAAG 5625

TAAATAAcGTTCTCTGATAAAGTTTAATCGGTCTTTTCCTATGTGGTTGGGTTGGCTTGTATACGGtGTAAGTTA 5700

aATACCAtgAGAgAATAAAcACCTCTTgaAGCaACgaatTAAAGGGTTTGTCATTCAATAAAACTAAGCcAGGAG 5775

AcAAGgCATTGtCAAAAGT 5850

Affolter, M., Percival-Smith, A., Müller, M., Leupin, W. and Gehring, W. J. (1990). DNA binding properties of the purified Antennapedia homeodomain. *Proc Natl Acad Sci U S A* **87**, 4093-4097.

Ebner, A., Cabernard, C., Affolter, M. and Merabet, S. (2005). Recognition of distinct target sites by a unique Labial/Extradenticle/Homothorax complex. *Development* **132**, 1591-1600.

Joshi, R., Passner, J. M., Rohs, R., Jain, R., Sosinsky, A., Crickmore, M. A., Jacob, V., Aggarwal, A. K., Honig, B. and Mann, R. S. (2007). Functional specificity of a Hox protein mediated by the recognition of minor groove structure. *Cell* **131**, 530-543.

Joshi, R., Sun, L. and Mann, R. (2010). Dissecting the functional specificities of two Hox proteins. *Genes Dev* **24**, 1533-1545.
